# Disrupted Functional Network Organization in Crohn's Disease: Interoceptive Alterations Linked to Anxiety

**DOI:** 10.1002/cns.71113

**Published:** 2026-08-30

**Authors:** Shuai Xu, Chunhui Bao, Xinyi Zhu, Qianfeng Wang, Boyu Zhang, Luyi Wu, Hongwei Li, Xuchen Yu, Zhou Hao, Zhensen Chen, He Wang, Huangan Wu

**Affiliations:** ^1^ Institute of Science and Technology for Brain‐Inspired Intelligence, Fudan University Shanghai China; ^2^ Human Phenome Institute, Fudan University Shanghai China; ^3^ Yueyang Hospital of Integrated Chinese and Western Medicine, Shanghai University of Traditional Chinese Medicine Shanghai China; ^4^ Shanghai Research Institute of Acupuncture and Meridian Shanghai China; ^5^ Department of Radiology Shanghai Fourth People's Hospital Affiliated to Tongji University School of Medicine Shanghai China; ^6^ Key Laboratory of Computational Neuroscience and Brain‐Inspired Intelligence (Fudan University) Ministry of Education Shanghai China

**Keywords:** anxiety, Crohn's disease, functional connectome, insula, interoception

## Abstract

**Background and Aims:**

How Crohn's disease alters large‐scale brain network organization and interacts with affective symptoms remains poorly understood. We aimed to characterize disease‐related disruptions in functional topology, rich‐club organization, and hierarchical gradients, and to determine their associations with disease activity and anxiety.

**Methods:**

Ninety‐seven Crohn's disease patients and 64 healthy controls underwent resting‐state fMRI. Graph‐theoretical metrics, rich‐club analysis, functional gradients, and spectral dynamic causal modeling were applied to assess network properties, hierarchical architecture, and effective connectivity. Associations with the Crohn's Disease Activity Index and anxiety were examined via linear regression.

**Results:**

Crohn's disease patients showed widespread network disruptions modestly linked to disease activity, including reduced global and local efficiency, impaired rich‐club connectivity, and compressed sensorimotor gradients. Decreased degree centrality in the insula, thalamus, and postcentral gyrus was accompanied by altered hierarchical organization and effective connectivity. Spectral dynamic causal modeling revealed anxiety‐related modulation of insular excitation and thalamo‐putaminal pathways.

**Conclusions:**

Crohn's disease involves widespread functional network disruption and reorganization, particularly in interoceptive regions supporting sensory and emotional processing. Anxiety modulates core limbic circuitry, highlighting the interplay between disease activity and affective symptoms in shaping brain network.

## Graphical abstract

Crohn's disease shows widespread disruption and reorganization of functional brain networks, with altered activity in core interoceptive regions involved in sensory and emotional processing. Emotional disturbances modulate brain network dynamics, providing insight into neural mechanisms linking disease activity and affective symptoms.

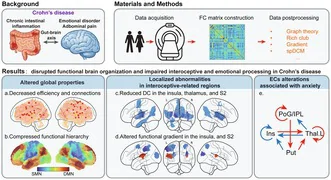

**Abbreviations**
AUC: Area under the curve
BMA: Bayesian model averaging
BMR: Bayesian model reduction
CD: Crohn's disease
CDAI: Crohn's disease activity index
CNS: Central nervous system
Cp: Clustering coefficient
CSF: Cerebrospinal fluid
DAN: Dorsal attention network
DC: Degree centrality
DMN: Default mode network
DSM‐IV: Diagnostic and Statistical Manual of Mental Disorders
ECN: Executive control network
ECs: Effective connections
Eg: Gobal efficiency
EIS: Extraintestinal symptoms
Eloc: Local efficiency
Ep: Expected posterior means
FA: Flip angle
FC: Functional connectivity
FDR: False discovery rate
FOV: Field of view
FuG: Fusiform gyrus
FWHM: Full‐width at half‐maximum
GABA+: Gamma‐aminobutyric acid
GE‐EPI: Gradient‐echo echo planar imaging
GM: Gray matter
GMV: Gray matter volume
HADS: Hospital Anxiety and Depression Scale
HCs: Healthy controls
IBD: Inflammatory bowel disease
IBDQ: Inflammatory bowel disease questionnaire
IPL: Inferior parietal lobule
ITG: Inferior temporal gyrus
Lp: Shortest path length
MCC: Middle cingulate cortex
MNI: Montreal Neurological Institute
mPFC: Medial prefrontal cortex
MRI: Magnetic resonance imaging
MVOcC: Medio ventral occipital cortex
PEB: Parametric empirical Bayes
PoG: Postcentral gyrus
Pp: Posterior probabilities
ReHo: Regional homogeneity
ROIs: Regions‐of‐interest
rs‐fMRI: Resting state functional magnetic resonance imaging
S2: Secondary somatosensory
SAD: Social anxiety disorder
SMN: Somatomoter network
SN: Salience network
spDCM: Spectral dynamic causal modeling
STG: Superior temporal gyrus
TE: Echo time
TPN: Temporo‐Parietal network
TR: Repetition time
VAN: Ventral attention network
VAS: Visual analog scale
VBM: Voxel‐based morphometry
VN: Visual network
WM: White matter

## 1 Introduction

Crohn's disease (CD) is a chronic gastrointestinal inflammatory disease and a major subtype of inflammatory bowel disease (IBD), which can affect the entire digestive tract from the oral cavity to the rectum [1]. Its prevalence is about 1% of the total population, and it has shown a rising trend in Asia in recent years [2]. CD is characterized by intestinal symptoms such as abdominal pain, diarrhea, and weight loss [3], as well as extraintestinal symptoms (EIS) involving multiple organ systems, including fatigue, anxiety, and depression [2]. Multi‐omics studies based on feces, blood, and other samples have revealed that impairment of the intestinal barrier serves as a crucial pathogenic factor in CD [1, 4]. Meanwhile, EIS in Crohn's disease, especially emotional symptoms, have also drawn growing attention [2, 5, 6, 7].

The gut‐brain axis, a central theoretical framework for understanding brain function and structure in CD, is a complex bidirectional communication system linking the central nervous system (CNS) and the gastrointestinal tract, facilitating interactions among the neural, endocrine, and immune systems [6, 7, 8]. Neuroimaging studies have consistently reported CD‐related alterations in brain function and structure. A meta‐review indicated that morphological and regional activity changes are primarily located within the default mode network (DMN) in remitted CD patients, a network involved in emotion regulation, decision‐making, and pain processing [9]. Bao et al. [10] reported significantly decreased regional homogeneity (ReHo) in the left insula and middle cingulate cortex (MCC), correlating with pain scores. Wang et al. [11] identified decreased gamma‐aminobutyric acid (GABA+) levels in the medial prefrontal cortex (mPFC), which may mediate associations between brain structural and functional alterations and depression in IBD. Disease‐activity‐specific analysis further revealed reduced gray matter volume (GMV) in sensorimotor, occipitotemporal, and medial frontal regions, with fatigue‐GMV correlations present only in remitted CD [12]. However, these findings are limited by small sample sizes and a predominant focus on localized brain regions.

Moreover, CD patients frequently experience higher rates of anxiety and depression compared with healthy individuals, with anxiety often presenting as a prominent and persistent symptom [11, 13, 14]. Mikocka–Walus et al. [15] found a strong association between depression/anxiety and clinical relapse of CD. Converging evidence suggests that peripheral inflammation can affect brain regions involved in reward, motor, emotional, and interoceptive processing [16, 17], including the basal ganglia, thalamus, and insula, highlighting the vulnerability of these neural circuits in inflammatory conditions [18]. Given the high prevalence of emotional disturbances, it is crucial to investigate how CD affects the neural systems supporting emotional regulation.

The human functional connectome provides a global perspective on brain organization patterns and is sensitive to both global and local alterations associated with disease states [19]. Advanced analytical approaches provide complementary insights into brain network organization. Graph‐theoretical metrics, including rich‐club organization, characterize the efficiency and robustness of information flow within brain networks [20, 21], while gradient analysis maps continuous hierarchical transitions from unimodal to transmodal processing [22]. Furthermore, spectral dynamic causal modeling enables the estimation of directed (effective) connectivity among regions [23]. Although these methods have been widely applied in neurological and psychiatric disorders [24, 25, 26], they have rarely been employed in CD research [27, 28], highlighting a gap in our understanding of emotion‐related neural alterations in CD patients.

Therefore, we first performed comprehensive connectome‐based analyses to characterize alterations in large‐scale functional organization in Crohn's disease. We then examined local functional and structural abnormalities and applied spectral dynamic causal modeling (spDCM) to characterize effective connectivity among these altered brain regions and their modulation by anxiety. Additionally, Crohn's Disease Activity Index (CDAI) and anxiety scores were incorporated to link network alterations with clinical symptoms. Together, these approaches provide a novel perspective on brain mechanisms in Crohn's disease and clarify how the disease interacts with emotional dysregulation.

## 2 Materials and Methods

### 2.1 Subjects

Ninety‐seven right‐handed CD patients were enrolled from the outpatient department of the Shanghai Research Institute of Acupuncture and Meridian and the Endoscopy Center of Zhongshan Hospital, Fudan University. All patients underwent systemic and gastrointestinal examinations and were diagnosed by an experienced gastroenterologist. Clinical measures included the Crohn's Disease Activity Index, the Inflammatory Bowel Disease Questionnaire (IBDQ), and the Visual Analog Scale (VAS) for abdominal pain. Anxiety and depression were assessed via the Hospital Anxiety and Depression Scale (HADS). Psychiatric disorders were excluded based on the Diagnostic and Statistical Manual of Mental Disorders (DSM‐IV). Inclusion criteria: (1) ≥ 6 years of education; (2) active/remission status for ≥ 6 months. Exclusion criteria: (1) CD‐related abdominal surgery history; (2) menstruating, pregnant, or lactating women; (3) use of corticosteroids, immunosuppressants, biological agents, or psychotropic drugs in the past 3 months; (4) metal implants, claustrophobia, or other magnetic resonance imaging (MRI) contraindications; (5) neurosurgery history or neurological disorders; (6) family history of psychiatric or neurological genetic diseases. Additionally, 64 right‐handed healthy controls (HCs) were recruited from Shanghai University of Traditional Chinese Medicine. Inclusion criteria: (1) ≥ 6 years of education; (2) no major/chronic disease; (3) no family genetic disease history; (4) no stimulant use in the past 3 months. Demographic information was recorded for all participants; detailed data are presented in Table 1.

**TABLE 1:** Demographic and clinical characteristics of CD patients and HCs.

	CD (*n* = 97)	HCs (*n* = 64)	*p*
Sex (male/female)	75/22	38/26	0.026*
Age (years)	32.0 ± 8.8	32.9 ± 8.5	0.490
Education (years)	16.2 ± 3.3	18.2 ± 3.4	< 0.001**
BMI	19.8 ± 2.8	—	—
Duration of CD (months)	54.6 ± 45.2	—	—
VAS maximum abdominal pain	2.7 ± 2.4	—	—
VAS average abdominal pain	2.0 ± 1.9	—	—
CDAI score	126.1 ± 72.9	—	—
IBDQ	170.3 ± 28.4	—	—
HADS‐A	6.0 ± 3.7	—	—
HADS‐D	4.7 ± 3.7	—	—

Abbreviations: BMI, body mass index; CDAI, Crohn's disease activity index; HAD‐A/HAD‐D, hospital anxiety and depression scale; IBDQ, inflammatory bowel disease questionnaire; VAS, visual analogue scale.

* Statistical significance at *p* < 0.05.

** Statistical significance at *p* < 0.01.

### 2.2 MRI Acquisition

MRI data were acquired using a 3.0 Tesla Siemens Verio scanner at the Shanghai Mental Health Center. Participants were instructed to lie still with their eyes closed and remain awake without engaging in any specific cognitive activity. High‐resolution T1‐weighted structural images were obtained with the following parameters: repetition time (TR) = 2300 ms, echo time (TE) = 2.98 ms, flip angle (FA) = 9°, slice thickness = 1.0 mm, field of view (FOV) = 256 mm × 256 mm, matrix size = 256 × 256, and 176 slices. Resting state functional magnetic resonance images (rs‐fMRI) were acquired using a gradient‐echo echo planar imaging (GE‐EPI) sequence with TR = 2000 ms, TE = 30 ms, FA = 90°, slice thickness = 5 mm, FOV = 240 mm × 240 mm, matrix size = 64 × 64, and 32 transverse slices.

### 2.3 T1‐Imaging Preprocessing

Voxel‐Based Morphometry (VBM) analysis was performed using the CAT12 toolbox (A Computational Anatomy Toolbox for SPM, https://neuro‐jena.github.io/cat/) [29] within SPM12 (Statistical Parametric Mapping version 12, https://www.fil.ion.ucl.ac.uk/spm/software/spm12/) [30] to investigate structural brain differences between CD patients and healthy controls. T1‐weighted images were preprocessed, including reorientation, segmentation into gray matter (GM), white matter (WM), and cerebrospinal fluid (CSF), and normalization to Montreal Neurological Institute (MNI) space using DARTEL (Diffeomorphic Anatomical Registration Through Exponentiated Lie Algebra). After smoothing with an 8 mm full‐width at half‐maximum (FWHM) kernel, statistical analyses were conducted to assess differences in GM volume, with a particular focus on subcortical regions.

### 2.4 Rs‐fMRI Preprocessing

The rs‐fMRI preprocessing was conducted using the DPABI toolkit (Data Processing & Analysis for Brain Imaging) [31] based on MATLAB 2021b (The MathWorks, Natick, MA). The preprocessing pipeline included the following steps: removal of the first ten volumes of resting‐state data; slice‐timing correction and head motion correction; rigid registration between structural and functional images, followed by normalization to MNI space; regression of white matter and ventricular signals as well as Friston 24 head motion parameters obtained from motion correction [32]; and bandpass filtering (0.01–0.1 Hz).

Participants were excluded if they met any of the following criteria: (1) image quality score below 3, (2) maximum translation exceeding 2 mm, (3) maximum rotation exceeding 2 degrees in any direction, or (4) mean framewise displacement (FD) exceeding 0.2 mm.

### 2.5 Rs‐fMRI Postprocessing

#### 2.5.1 Graph‐Theoretical Analysis

The GRETNA toolkit (Graph Theoretical Network Analysis toolkit) [33] was used to perform graph theoretical analysis of rs‐fMRI. Two atlases were combined to create Parcellation 1: the Brainnetome atlas [34] and the SUIT atlas (a spatially unbiased atlas template of the cerebellum and brainstem) [35, 36, 37] covering 280 brain regions in total. Functional connectivity network matrices were constructed by calculating Pearson correlations between the time series of all pairs of brain regions, followed by applying Fisher's r‐to‐z transformation to the resulting matrices. A series of global network and node properties were calculated, including global efficiency (Eg), local efficiency (Eloc), shortest path length (Lp), clustering coefficient (Cp), and node degree centrality (DC). For each measure, the area under the curve (AUC) was obtained across sparsity thresholds ranging from 0.05 to 0.4 (step = 0.01).

#### 2.5.2 Rich‐Club Analysis

Rich‐club analysis was performed based on the functional connectivity network using a 10% sparsity threshold. For each participant, the rich‐club coefficient *φ* (*k*) was computed, where *k* represents the degree range (1–40 in this study) [38]. The normalized rich‐club coefficient *φ*_norm_ (*k*) was obtained by dividing *φ* (*k*) of the real network by the mean *φ* (*k*) derived from 1000 randomly rewired networks. The number of remaining nodes and edges at each *k* level was recorded. Hubs (top 16% degree) were identified, and rich‐club, feeder, and local connection strengths, along with inter‐hub Euclidean distance, were calculated for statistical comparisons.

#### 2.5.3 Connectome Gradient Analysis

Gradient analysis was performed using the BrainSpace Toolbox (https://github.com/MICA‐MNI/BrainSpace) [39]. Functional connectivity matrices (495 × 495) were constructed by integrating three atlases: the Schaefer's 400‐parcel 17‐network atlas [40], the Tian subcortical atlas [41], and the SUIT cerebellar atlas [35, 36, 37], named as Parcellation2. The resulting matrices were subjected to Fisher's Z transformation. The multi‐atlas strategy was adopted to yield a larger number of brain regions with comparable size, enabling more precise gradient estimation [39]. For each matrix, the top 10% of connections per row were retained, and a normalized angle similarity matrix was computed. A diffusion embedding method was then applied for dimensionality reduction to obtain gradient components. The component associated with the largest eigenvalue was selected as the primary gradient. To enhance comparability across participants, gradient components were further aligned using Procrustes transformation.

#### 2.5.4 Spectral Dynamic Causal Modeling Analysis

Spectral dynamic causal modeling was used to estimate effective connectivity within the interoceptive network. Regions‐of‐interest (ROIs), including the insula, putamen, thalamus, postcentral gyrus (PoG), and inferior parietal lobule (IPL), were defined a priori. For each participant, a fully connected spDCM model was specified using ROI‐averaged time series, assuming intrinsic coupling without external inputs. Model inversion was performed using cross‐spectral density to infer effective (directed) connectivity parameters. At the group level, individual connection parameters were entered into a Parametric Empirical Bayes (PEB) model examining the effects of anxiety, CDAI, and their interaction, controlling for age, sex, and years of education. Bayesian model reduction (BMR) using a greedy search procedure was applied to prune redundant parameters, followed by Bayesian model averaging (BMA) to obtain posterior estimates of effective connectivity. Results are reported as expected posterior means (Ep) with posterior probabilities (Pp).

### 2.6 Statistical Analysis

#### 2.6.1 Comparative Analysis

Between‐group differences were assessed using two‐sample *t*‐tests or Mann–Whitney U tests based on data distribution. Voxel‐wise GMV analysis was performed using *p* < 0.001 (uncorrected) with a cluster‐level false discovery rate (FDR) correction at *q* < 0.05. Region‐wise analyses applied FDR correction (*q* < 0.05). All statistical models included age, sex, years of education, and mean FD as covariates. For subnetwork‐level gradient analysis, brain regions were grouped into ten predefined functional networks. Mean gradient values were calculated for each participant and compared between groups, with FDR correction applied across subnetworks (*q* < 0.05).

#### 2.6.2 Correlation and Linear Regression Analysis

Correlation analyses were performed between CDAI and multiple network metrics, including global properties, nodal properties, the rich‐club coefficient, and rich‐club connection strength.

The DPABI toolbox was used to calculate DC by thresholding correlations at *r* > 0.25 between each voxel and all other voxels in the brain. The resulting DC maps were standardized using *z*‐scores and smoothed with a 6 mm Gaussian kernel. A multiple regression model was applied to examine the associations between DC maps and CDAI. A full factorial model was used to analyze CDAI and HAD‐A interaction effects on DC, with the same covariates as described above. All significant clusters were thresholded at *p* < 0.001 and cluster‐level FDR correction at *q* < 0.05.

### 2.7 Sensitivity Analyses

Robustness analyses evaluated the dependency of our results on atlas choice and demographic factors. We repeated graph‐theoretical analyses using parcellation2 (495 regions) in the main sample and performed sex‐stratified and demographically matched subsample analyses with the original pipeline (see Sensitivity Analyses S1).

## 3 Results

### 3.1 Demographic and Clinical Data

The demographic and clinical data of CD patients and healthy controls are summarized in Table 1. Compared with HCs, CD patients had a significantly higher percentage of males (*p* = 0.026) and fewer years of education (*p* < 0.001). No significant difference in age was observed between the two groups (*p* = 0.490).

### 3.2 Impaired Global Properties of the Functional Connectome Associated With CDAI

**FIGURE 1:**
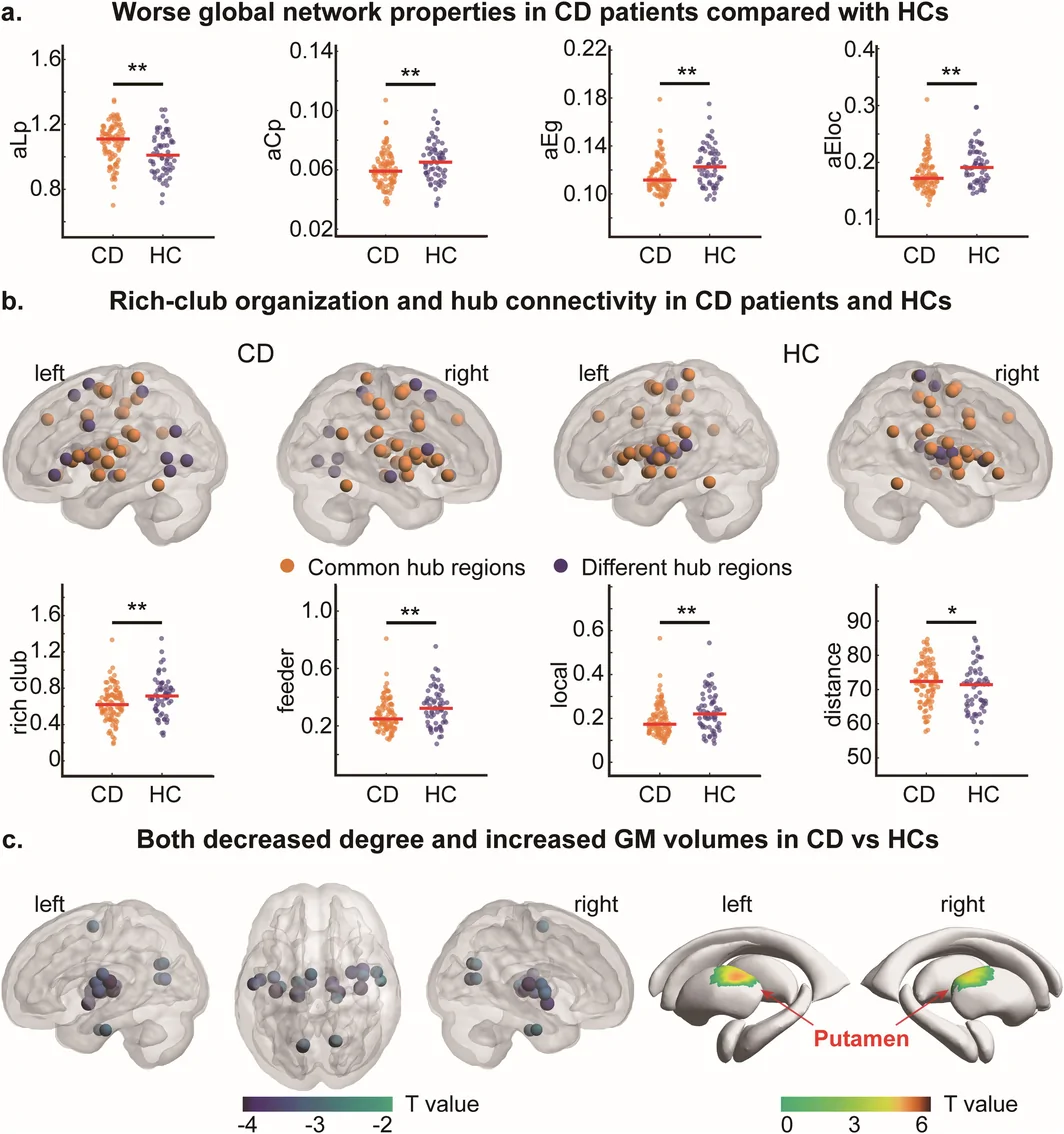
Altered brain network properties and regional changes in CD patients compared with HCs. (a) CD patients showed decreased global efficiency, local efficiency, and clustering coefficient, along with increased shortest path length, indicating impaired global functional network properties. (b) Group differences in hub region distribution. Compared with HCs, CD patients exhibited reduced average connection strength across all connection types and increased average distance between hub regions. Rich‐club connections refer to connectivity between hub regions, feeder connections to connectivity between hub and non‐hub regions, and local connections to connectivity between non‐hub regions. (c) CD patients showed decreased degree mainly in the bilateral insula and thalamus and increased GMV in the bilateral putamen. All results except for GMV survived FDR correction at *q* < 0.05 (*indicates *q* < 0.05; **indicates *q* < 0.01). GMV results were thresholded at *p* < 0.001 with cluster‐level FDR correction at *q* < 0.05. aCp, AUC of clustering coefficient; aEg, AUC of global efficiency; aEloc, AUC of local efficiency; aLp, AUC of shortest path length; AUC, area under the curve; GMV, Gray matter volume.

Small‐world properties were evident in the functional networks of both CD patients and HCs. Compared with HCs, CD patients showed significantly lower global efficiency, local efficiency, and clustering coefficient, as well as a significantly higher global shortest path length (FDR‐corrected, *q* < 0.05). These results indicate a disrupted global topological organization of the functional connectome in CD patients (Table 2, Figure 1). After controlling for age, sex, years of education, and mean FD, modest but significant correlations between CDAI and the global properties of the functional connectome were observed (FDR‐corrected *q* < 0.05; Table 2).

**TABLE 2:** Significant differences in global properties and rich club between CD patients and HCs, and correlations with CDAI.

CD versus HCs	*t* value	FDR‐*q* value	Correlation with CDAI	
			*R* value	FDR‐*q* value
Global properties measure the overall structural characteristics of a functional network				
Shortest path length	3.59	0.001^**^	0.22	0.038^*^
Clustering coefficient	−2.65	0.009^**^	−0.19	0.038^*^
Network efficiency	−3.46	0.001^**^	−0.21	0.038^*^
Network localefficiency	−3.44	0.001^**^	−0.19	0.038^*^
Rich‐club indicates a network's subset of highly interconnected nodes				
Rich‐club connections	−2.77	0.008^**^	−0.17	0.069
Feeder connections	−2.90	0.008^**^	−0.24	0.044^*^
Local connections	−3.00	0.008^**^	−0.21	0.045^*^
Distance among hub regions	2.44	0.016^*^	0.04	0.349

*Note:* All results were corrected using FDR at *q* < 0.05. Rich‐club connections refer to connections between hub regions; feeder connections refer to connections between hub and non‐hub regions; local connections refer to connections between non‐hub regions.

* Significant at *q* < 0.05.

** Significant *q* < 0.01.

### 3.3 Altered Rich‐Club Organization of the Functional Connectome in Crohn's Disease

Differences in hub distribution were observed between CD patients and HCs, with CD patients showing a greater mean inter‐hub distance compared with HCs (Figure 1, Table 2, and Table S1). Connectivity strength was found to be significantly reduced in CD patients relative to HCs across rich‐club, feeder, and local connections (FDR‐corrected *q* < 0.05). In addition, feeder and local connectivity strengths were negatively correlated with CDAI. Additional details are provided in Table S2 and Figure S1.

### 3.4 Gradient Changes of the Functional Connectome

**FIGURE 2:**
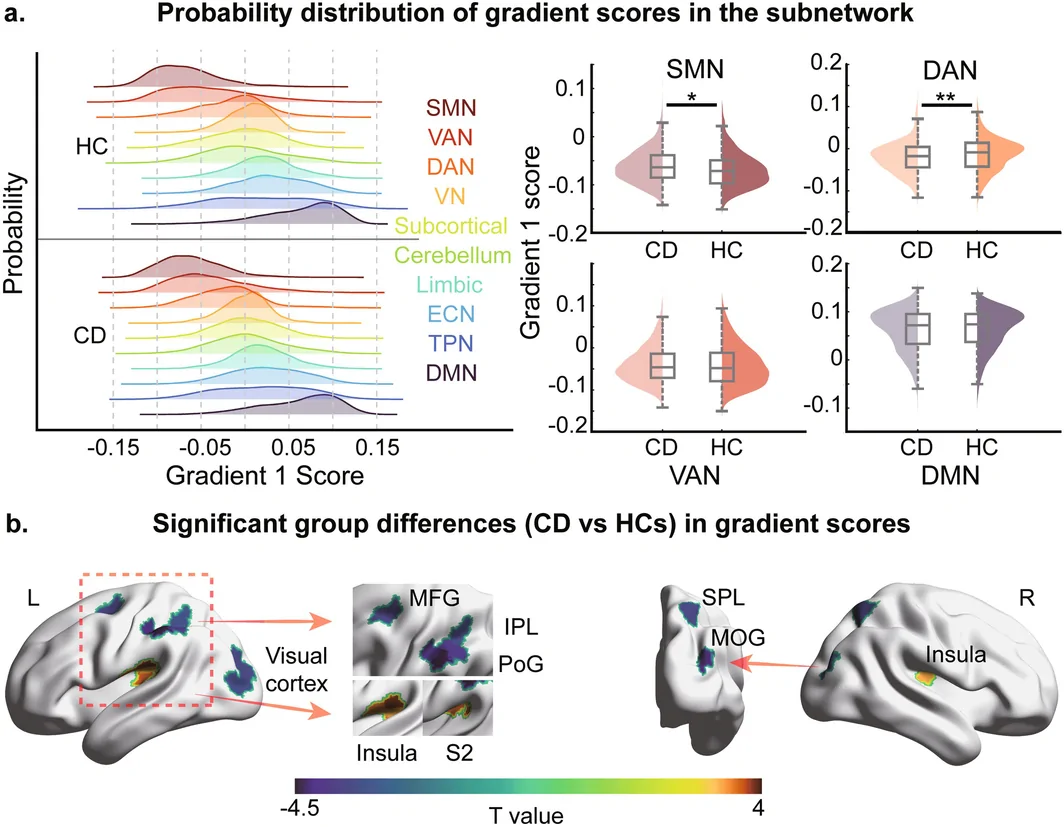
Altered hierarchical organization in CD patients. (a) Probability distributions of gradient scores in CD patients and HCs revealed a similar principal axis from the SMN to the DMN. However, CD patients showed significant alterations in the probability distribution at the lower end of the axis, involving networks such as the SMN and dorsal DAN. (b) Significant group differences in gradient scores between CD patients and HCs. Red areas indicate increased gradient scores in CD patients (left insula, left S2), whereas blue areas indicate decreased gradient scores (left postcentral gyrus). All results survived FDR correction at *q* < 0.05 (*indicates q < 0.05; **indicates q < 0.01). DAN, Dorsal attention network; DMN, Default mode network; ECN, Executive control network; IPL, Inferior parietal lobule; MFG, Middle frontal gyrus; MOG, Middle occipital gyrus; PoG, Postcentral gyrus; S2, secondary somatosensory cortex; SMN, Somatomotor network; SPL, Superior parietal lobule; TPN, Temporo‐Parietal network; VAN, ventral attention network; VN, Visual network.

In this study, only the first principal gradient (G1) explained more than 15% of the variance in the functional connectome. This principal gradient exhibited a continuous axis in both groups, with the sensory system located at one extreme and the transmodal DMN at the other. Notably, the gradient in CD patients showed a unilateral compression pattern at the sensory end of the axis. Further group comparisons revealed that CD patients showed significantly increased gradient scores in the bilateral insular cortex and secondary somatosensory (S2) cortex, whereas significantly decreased scores were observed in the left extrastriate cortex of the visual network, as well as in the left PoG, left IPL and the right parietal occipital area of the dorsal attention network (DAN) (FDR‐corrected, *q* < 0.05). Detailed results are presented in Figure 2; Figure S2, and Tables S3 and S4.

### 3.5 Increased GMV in Bilateral Putamen in CD Patients

Compared with HCs, significantly increased GMV was observed in the bilateral putamen of CD patients (*p* < 0.001, cluster‐level FDR‐corrected *q* < 0.05). Detailed results are presented in Table 3 and illustrated in Figure 1.

**TABLE 3:** Significantly increased GMV and significant correlation & interaction effects between DC, CDAI, and HAD‐A in CD patients.

CD versus HCs	Centroid			Cluster size	*t* value	FDR‐*q* value
	X	Y	Z			
Significant increased GMV in subcortical nuclei						
Putamen_L	−30	−8	3	606	5.55	0.005**
Putamen_R	28	−12	6	595	5.32	0.005**
Significant negative effects of CDAI on DC						
Putamen_L	−21	6	−3	53	−4.57	0.032*
	−21	−3	6		−3.82	
	−21	9	6		−3.47	
Putamen_R	27	3	−3	52	−3.88	0.032*
	24	6	6		−3.80	
	24	6	15		−3.29	
Significant interaction effects between CDAI and HAD‐A in DC						
Extending from Parietal_Inf_L to Postcentral_L and Precentral_L	−36	−39	54	90	4.73	0.011*
	−27	−48	63		4.39	
	−15	−54	72		4.27	

*Note:* All results were thresholded at *p* < 0.001, cluster‐level FDR‐corrected at *q* < 0.05. *q < 0.05; **q < 0.01.

Abbreviation: Parietal_Inf, inferior parietal lobule.

### 3.6 Decreased DC Associated With CDAI and Anxiety in CD Patients

Compared with HCs, CD patients showed significantly decreased DC (FDR‐corrected, *q* < 0.05) in several brain regions, including the left precuneus, left postcentral gyrus, bilateral insula, bilateral thalamus, left superior temporal gyrus (STG), right inferior temporal gyrus (ITG), bilateral fusiform gyrus (FuG), and bilateral medioventral occipital cortex (MVOcC). Detailed information is provided in Figure 1 and Table S3.

**FIGURE 3:**
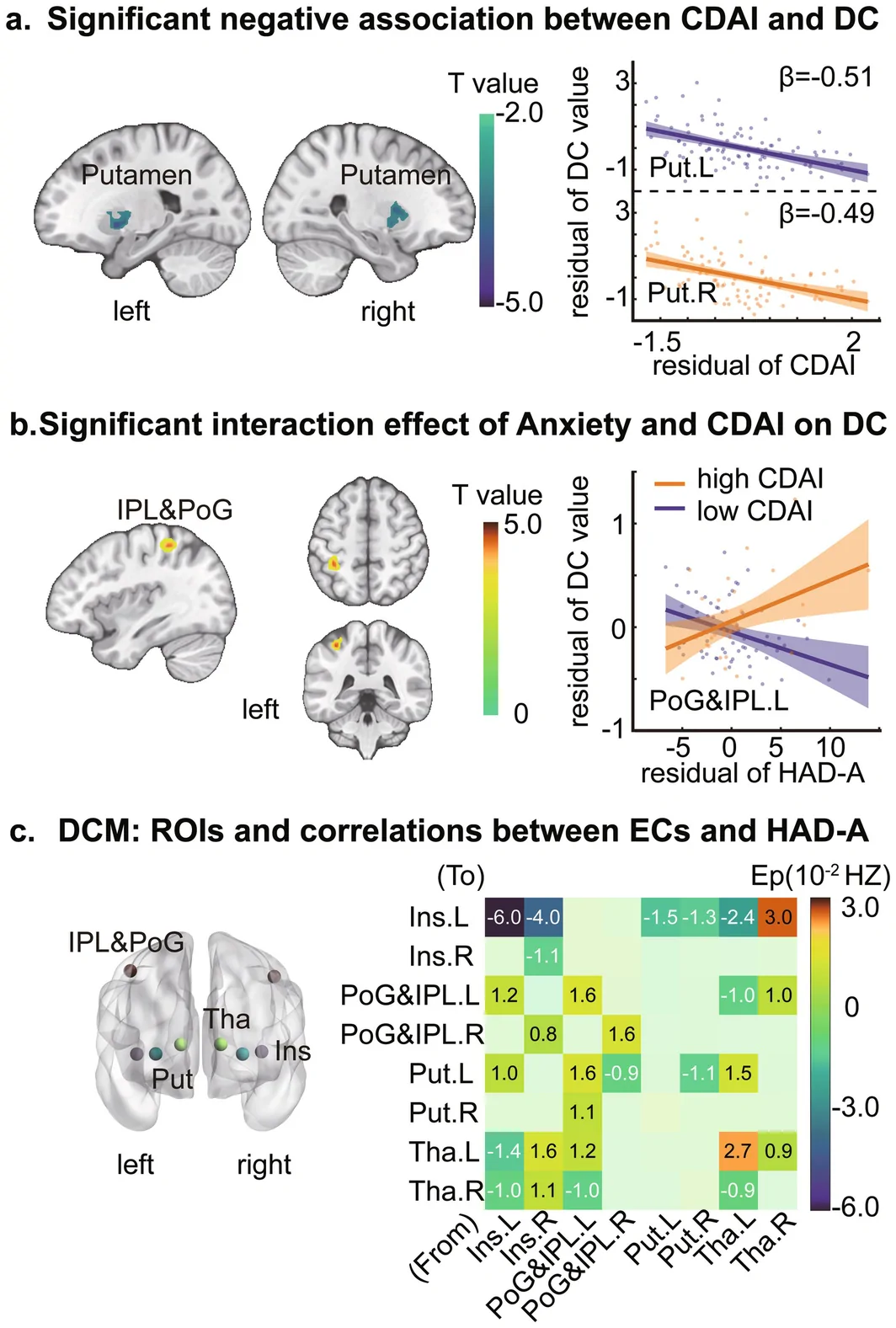
Associations between CDAI, anxiety, and effective connectivity in CD patients. (a) Significant negative associations were observed between CDAI and DC in the bilateral putamen. The right panel shows scatter plots of the linear regression between CDAI and DC in the bilateral putamen. (b) Significant interaction between anxiety and CDAI on DC was identified in the left IPL and left PoG. The right panel shows scatter plots of the regression between anxiety and DC in the left IPL and left PoG in CD patients with different CDAI grades. All results in panels a, b were significant at cluster‐level FDR‐corrected at *q* < 0.05, with an initial voxel‐level threshold of *p* < 0.001. (c) ROIs, mainly selected from key nodes of the interoceptive network, were used for spectral dynamic causal modeling analysis. Correlations between EC and anxiety are shown, thresholded at *p* > 0.99. CDAI, Crohn's Disease Activity Index; DC, Degree centrality; EC, Effective connectivity; Ep, Expected posterior; IPL, Inferior parietal lobule; PoG, Postcentral gyrus; P*p*, posterior probability; ROIs: Regions of interest.

In CD patients, the DC values of the bilateral putamen were significantly negatively correlated with CDAI (*p* < 0.001, cluster‐level FDR‐corrected *q* < 0.05). The DC in the left postcentral gyrus and left inferior parietal lobule showed a significant interaction effect between CDAI and anxiety (HAD‐A). Detailed results are presented in Figure 3, Table 3 and Table S5.

### 3.7 Altered Effective Connectivity Associated With Anxiety in Key Interoceptive Brain Regions

After controlling for age, sex, years of education, and mean FD, 28 altered effective connections (ECs) associated with anxiety (HAD‐A) were identified (Figure 3). Detailed significant results are presented in Table S6.

### 3.8 Sensitivity Analyses

The main findings were robust to alternative parcellation (Tables S7 and S8) and to sex‐stratified and matched subsample analyses (Tables S9–S11; Figures S3 and S4), with consistent effect directions and core group differences.

## 4 Discussion

This study characterizes large‐scale brain network alterations in Crohn's disease. Widespread topological and rich‐club disruptions showed modest but significant links to disease activity. Functional hierarchical organization was also disrupted, marked by a contracted gradient within low‐level sensory systems. Regionally, reduced degree and abnormal functional gradients were found in key sensory and interoceptive areas (insula, postcentral gyrus, thalamus, and putamen). These regional alterations also showed associations with disease activity and with anxiety, suggesting that disease activity and affective symptoms may share common neural substrates within interoceptive and limbic circuits. Together, these findings support a network‐level neurobiological framework in which systemic disease burden and affective state jointly shape central representation of Crohn's disease, providing insight into potential mechanisms underlying symptom modulation along the gut‐brain axis.

Widespread disruption and reconfiguration of large‐scale brain functional network organization were detected in CD patients. Analyses of global topological properties and rich‐club organization revealed reduced global efficiency and disrupted network architecture, both modestly correlated with disease activity (CDAI). Specifically, decreases in global and local efficiency, together with increased characteristic path length, indicate impaired network integration and communication efficiency [42, 43]. These findings align with previous reports in Crohn's disease [27, 28], supporting large‐scale neural dysfunction. Furthermore, a sparser distribution of hub regions, reduced connectivity among rich‐club, feeder, and local connections, a lower rich‐club coefficient (Φ), and fewer edges among high‐degree nodes (*k* > 28) suggest that the brain's core integrative architecture is particularly vulnerable to disease‐related disruption. The associations between these network alterations and CDAI further reinforce the link between inflammation and impaired functional integration. However, the modest effect sizes suggest that brain network reorganization in CD likely reflects the combined influence of inflammation, chronic pain, fatigue, and psychological factors not fully captured by CDAI alone [44].

Beyond global topological disruption, gradient analysis revealed an altered hierarchical organization of the functional network in CD. A canonical functional gradient axis was identified in both groups, with the lower‐order sensory system at one end and the transmodal default mode network at the other, consistent with a hierarchical organization from unimodal to transmodal processing across the cortex [22]. Compared with HCs, CD patients exhibited a contracted gradient distribution within the low‐level sensory system, indicating reduced functional separation between sensory and transmodal networks. This pattern of selective gradient compression in the sensorimotor system, which reduces network differentiation and disrupts somatosensory‐motor organization as well as the integration of bottom‐up sensory information with higher‐order attentional processes, parallels findings in schizophrenia where low‐order sensory gradient compression has been linked to attentional and perceptual deficits [45].

Regionally, decreased DC and altered G1 scores were observed in key interoceptive hub regions involved in processing and interpreting internal bodily signals [16]. Reduced DC was primarily found in the bilateral insula, bilateral thalamus, bilateral precuneus, and left PoG, indicating decreased global functional integration. Increased gradient scores were observed in the somatomotor network (bilateral S2 and insula), suggesting altered hierarchical organization and enhanced local functional integration. These regions form core hubs of the pain matrix involved in processing sensory, emotional, and interoceptive information [16, 46, 47, 48]. Decreased ReHo in the left insula has been reported in CD patients with abdominal pain [9, 10]. The apparent dissociation between reduced DC and increased gradient scores may reflect compensatory functional reorganization, whereby regions with diminished global connectivity enhance hierarchical integration to maintain efficient interoceptive and sensorimotor processing in CD. Notably, bilateral putamen showed increased GMV and significant negative correlations between DC and CDAI, suggesting altered basal ganglia integration within cortico‐basal ganglia‐thalamo‐cortical circuits. Together, these findings indicate disrupted hub integrity and reorganization of interoceptive–sensorimotor networks, linking disease severity to impaired large‐scale and subcortical communication.

Moreover, CD patients showed disrupted emotion‐interoception coupling, suggesting that anxiety may modulate disease‐related neural reorganization. Emotional dysregulation, particularly anxiety, is a common comorbidity in CD and closely linked to symptom severity and pain perception [12, 49, 50]. Sun et al. reported that CD patients with anxiety or depression exhibited reduced functional connectivity (FC) between the left superficial subregion of the amygdala and the left insula [51], consistent with insular alterations in our study. Gradient analyses further revealed reduced differentiation within the dorsal attention network (PoG, IPL) and visual network, indicating impaired hierarchical organization in systems supporting attention and sensory integration. Significant CDAI × anxiety interactions on DC in PoG and IPL highlight these somatosensory regions as key nodes where inflammatory burden and emotional dysregulation jointly shape network communication. The putamen is involved in reward, motivation, emotion, and reinforcement learning [52, 53], and may link disrupted interoceptive integration to higher‐order emotional regulation. Supporting this interpretation, spDCM analyses demonstrated anxiety‐related reorganization of the insula‐thalamus‐putamen pathway. Higher anxiety scores were associated with reduced self‐inhibition of the bilateral insula and weakened left thalamo‐insular and putamino‐insular influences, suggesting increased insular excitability and diminished regulatory control, which may enhance sensitivity to interoceptive and affective signals and contribute to heightened pain perception and emotional reactivity [48]. Notably, thalamo‐insular connectivity showed lateralized reorganization: left‐thalamic influence on the left insula was reduced, whereas right‐thalamic influence was enhanced. This was accompanied by stronger right insula‐to‐thalamus coupling, suggesting asymmetric thalamocortical reorganization. Anxiety also strengthened left thalamic and PoG/IPL inputs to the putamen, indicating enhanced integration of somatosensory and interoceptive information within basal ganglia circuits, facilitating salience processing and sensorimotor readiness [16, 54, 55]. Increased self‐inhibition of the left thalamus with reduced thalamic coupling to PoG/IPL may further reflect diminished thalamocortical transmission and altered sensory gating, indicating that anxiety shifts information processing away from conventional thalamocortical pathways toward putaminal circuits involved in emotional and behavioral adaptation [56]. Collectively, these patterns provide a mechanistic account for disrupted coupling between emotion and bodily signal processing in CD, explaining how anxiety exacerbates symptom perception and disease burden.

In summary, this study provides novel evidence that Crohn's disease is characterized by multi‐scale functional network reorganization, including impaired network integration, compressed hierarchical gradients, and disrupted interoceptive‐subcortical circuits. Critically, we demonstrate for the first time that anxiety modulates effective connectivity within the insula‐thalamus‐putamen pathway, offering a causal mechanism linking emotional dysregulation to heightened symptom perception. These findings reveal a multi‐level framework for understanding gut‐brain interactions in CD, highlighting the insula as a key node where peripheral disease activity, interoceptive processing, and affective dysfunction converge. Future studies should incorporate structural covariance networks [57] and structural‐functional network coupling analyses [58] to clarify whether observed functional reorganization is driven by large‐scale structural remodeling, providing a more comprehensive view of disease‐related neural alterations.

## 5 Limitations

Several limitations should be acknowledged. First, the significant sex imbalance between CD patients and HCs may introduce residual confounding despite covariate adjustment. Second, the absence of gut microbiota data precludes direct examination of brain‐gut interactions. Third, brain functional alterations likely vary with disease activity, warranting future subgroup analyses. Fourth, the limited sample size and varying disease duration prevented stratification by disease duration. Fifth, excluding patients on corticosteroids, immunosuppressant's, or biologics, while necessary, limits our findings to the milder, untreated CD spectrum.

## 6 Conclusions

In conclusion, the present findings establish a multi‐level framework for understanding gut‐brain interactions in Crohn's disease, revealing that functional network reorganization—particularly within the insula, thalamus, and basal ganglia—serves as a neural substrate linking peripheral disease activity to symptom perception and emotional dysregulation. Critically, the demonstration that anxiety causally modulates the insula‐thalamus‐putamen pathway provides a mechanistic link between affective dysfunction and heightened symptom burden. Together, these results suggest that targeting anxiety and emotion‐regulatory circuits may offer a novel therapeutic avenue for alleviating both symptom perception and affective distress in CD.

## Author Contributions

Shuai Xu: conceptualization; methodology; formal analysis; writing – original draft; writing – review and editing. Chunhui Bao: data curation; conceptualization; methodology; funding acquisition; writing – review and editing. Xinyi Zhu: data curation; investigation; formal analysis. Qianfeng Wang: writing – review and editing. Boyu Zhang: writing – review and editing. Luyi Wu: data curation; conceptualization; funding acquisition. Hongwei Li: writing – review and editing. Xuchen Yu: writing – review and editing. Zhou Hao: data curation; investigation; formal analysis. Zhensen Chen: writing – review and editing. He Wang: conceptualization; supervision; project administration; funding acquisition; writing – review and editing. Huangan Wu: conceptualization; project administration.

## Funding

This work was supported by the 2024 and 2025 Shanghai Oriental Talent Plan Youth Project; the Key Research Laboratory of Acupuncture and Immune Effects of the National Administration of Traditional Chinese Medicine; the Special Clinical Research Project in the Health Industry of Shanghai Municipal Health Commission [No. 202340036]; the Shanghai Rising‐Star Program [No. 19QA1408100]; the National Natural Science Foundation of China [Nos. 82271956, 62331021 and 82422074 (Excellent Youth Fund)]; the Shanghai Municipal Science and Technology Explorer Project [No. 23TS1400500]; the National Key Research and Development Program of China [No. 2023YFF1204804].

## Ethics Statement

This study has obtained approval from the Ethics Committee of Yueyang Hospital of Integrated Traditional Chinese and Western Medicine, affiliated with Shanghai University of Traditional Chinese Medicine (Approval No. 2024‐228). All participants have signed informed consent forms. The study protocol was in compliance with the principles of the Declaration of Helsinki.

## Conflicts of Interest

The authors declare no conflicts of interest.

## Supporting information


**Figure S1:** Rich‐club across *k*‐levels in CD patients and HCs.
**Figure S2:** Group‐averaged principal gradient and global/subnetwork gradient distributions in CD patients and HCs.
**Figure S3:** Cross‐sample replication of degree centrality group differences.
**Figure S4:** Cross‐sample replication of Gradient 1 scores group differences.

**Sensitivity Analysis S1:** Validation of group differences against methodological and demographic.

**Table S1:** Hub regions in CD patients and HCs.
**Table S2:** Significant differences in Rich‐club properties between CD and HCs.
**Table S3:** Significant differences in nodal DC and G1 between CD and HCs.
**Table S4:** Significant differences in distribution of gradient scores in Sub‐networks between CD patients and HCs.
**Table S5:** Significant effective connectivity associated with anxiety (HAD‐A) in CD patients.
**Table S6:** Post hoc linear regression and interaction analyses of DC values in CD patients.
**Table S7:** Significant differences in global properties and rich club between CD patients and HCs based on Parcellation 2 (final analytic sample, *n* = 161).
**Table S8:** Significant differences in nodal DC between CD and HCs based on Parcellation 2 (final analytic sample, *n* = 161).
**Table S9:** Demographic and clinical characteristics of CD patients and HCs in the demographically matched subsample (*n* = 122).
**Table S10:** Sensitivity analyses of global properties and rich‐club organization: sex‐stratified subsamples (males, *n* = 113; females, *n* = 48) and demographically matched subsample (*n* = 122), based on Parcellation 1.
**Table S11:** Sensitivity analyses of AUC of DC and Gradient score 1: sex‐stratified subsamples (males, *n* = 113; females, *n* = 48) and demographically matched subsample (*n* = 122).

## Data Availability

The data underlying this article will be shared on reasonable request to the corresponding author.
