# Supplementary material for: Disrupted Functional Network Organization in Crohn's Disease: Interoceptive Alterations Linked to Anxiety
Source: CNS Neurosci Ther. 2026 Aug 30;32(9):e71113. doi: 10.1002/cns.71113 (PMC13526404; doi:10.1002/cns.71113)
Supplement: Supplementary file 1 [file CNS-32-e71113-s001.docx]

**Supplementary Sensitivity Analysis**

**1. Methods**

All analytical procedures—including MRI preprocessing^1^, network construction, graph theory metrics calculation, gradient 1 score calculation, and statistical comparisons—were conducted identically to those described in the main manuscript Methods, except for the specific variations detailed below. No novel methodologies are introduced. Graph-theoretical analyses were carried out using the GRETNA toolbox^2^ including clustering coefficient^3^, shortest path length^3^, network efficiency^4^, network local efficiency^4^, nodal degree centrality (DC)^5^, and rich club metrics (average rich club connection strength, average feeder connection strength, average local connection strength, and mean distance of hub regions)^6^. Gradient analysis was performed with the BrainSpace toolbox^7^. For group comparisons, two-sample t-tests were employed, with age, sex, years of education, and mean framewise displacement (FD)^8^ included as covariates.

1.1 **Alternative Parcellation (Graph-theoretical validation)**

The primary graph-theoretical analysis utilized a 280-region parcellation (Parcellation 1: Brainnetome cortical atlas^9^ + SUIT cerebellar atlas^10-12^). To ensure the reproducibility of our graph-theoretical findings under the parcellation scheme used for the gradient analysis, we repeated the graph-theoretical analyses using a 495-region parcellation (Parcellation 2). This comprised the Schaefer 2018 400-region cortical parcellation (17 networks)^13^, the Tian subcortical atlas^14^, and the SUIT cerebellar atlas^10-12^. For the network construction, network sparsity thresholds were maintained within the same range (5%-40%, step 0.01) as the main analysis to ensure comparability of small-world^3^ architecture across parcellations.

**1.2 Demographic Subgroup and Matched-Sample Analyses**

To assess the influence of demographic factors, we performed two additional sets of analyses. (1) Post-hoc sex-stratified analyses: The main sample was divided into male (n=113) and female (n=48) subgroups. Given the reduced sample size in the female subgroup, these analyses were conducted as post-hoc exploratory validations rather than full discovery analyses. Specifically, two-sample t-tests were applied exclusively to the regions and graph metrics that survived the main between-group comparisons. (2) Demographically matched subsample: To further isolate the effect of the primary grouping variable from potential influences of sex and education, we performed propensity score matching (PSM)^15^ using a logistic regression model with sex and years of education as covariates to estimate propensity scores. A 1:1 nearest-neighbor matching algorithm (without replacement) was then applied, with a caliper width set to 1.0 standard deviation of the logit-transformed propensity score. The procedure yielded a well-balanced subsample of 122 participants (61 per group), with no significant between-group differences in sex, age, or years of education (all p > 0.05; Supplementary Table 9). Subsequently, both the graph-theoretical (Parcellation 1) and gradient (Parcellation 2) pipelines were completely rerun on this matched cohort, strictly adhering to the same analytical protocols as the main analyses.

**2. Results**

**2.1 Robustness of Graph-Theoretical Metrics to Parcellation Scheme**

To verify that the observed group differences in small-world topology were not driven by the specific nodal definition, we recalculated all graph metrics using the alternative 495-region parcellation (Parcellation 2). Consistent with the main findings, the CD patients exhibited significantly lower clustering coefficient, network efficiency, network local efficiency, and higher shortest path length compared to HCs (FDR-corrected, q<0.05; Supplementary Table 7). Beyond these global topological alterations, the CD group exhibited significant nodal DC decreases relative to HCs, and the spatial distribution of these nodes was highly concordant with that observed in the main analysis (FDR-corrected, q<0.05; Supplementary Table 8).

**2.2 Sex-Stratified Analyses**

The post-hoc sex-stratified analyses were conducted in male (n=113) and female (n=48) subgroups. In the male subgroup, significant between-group differences were observed in nodal DC (based on Parcellation 1) and gradient 1 scores (based on Parcellation 2) in a subset of regions that overlapped with those identified in the main analysis (FDR-corrected, q<0.05). However, none of the global graph-theoretical metrics and rich club metrics reached statistical significance in the male subgroup. In the smaller female subgroup, no metrics survived FDR correction. Critically, the directionality of all group differences (both global and nodal) remained consistent with the main findings across both sex subgroups (see Supplementary Tables 10–11, Supplementary Figures 3–4), suggesting that the reduced significance in the female subgroup is likely attributable to limited statistical power rather than a genuine absence of effects.

**2.3 Validation in the Demographically Matched Subsample**

We reran the entire analytical pipeline in a demographically matched subsample (n=122, 61 per group) obtained via propensity score matching. In this matched cohort, all global graph-theoretical metrics and rich club metrics showed significant group differences consistent with the main results (FDR-corrected, q<0.05). For nodal metrics, the CD group exhibited significant nodal DC reductions compared to HCs in spatially concordant regions with the main analysis—most notably in the insula and bilateral thalamus (FDR-corrected, q<0.05). Additionally, significant gradient 1 score decreases were observed in the left postcentral gyrus (FDR-corrected, q<0.05). Although only a subset of the originally significant nodal and gradient regions survived FDR correction in this matched sample, all effect directions remained fully aligned with the primary analysis (see Supplementary Tables 10–11, Supplementary Figures 3–4), further corroborating the robustness of our core conclusions against demographic confounders.

**References**

1.	Yan CG, Wang XD, Zuo XN, Zang YF. DPABI: Data Processing & Analysis for (Resting-State) Brain Imaging. *Neuroinformatics.* 2016;14(3):339–351.

2.	Wang JH, Wang XD, Xia MR, Liao XH, Evans A, He Y. GRETNA: a graph theoretical network analysis toolbox for imaging connectomics. *Frontiers in Human Neuroscience.* 2015;9.

3.	Watts DJ, Strogatz SH. Collective dynamics of 'small-world' networks. *Nature.* 1998;393(6684):440–442.

4.	Latora V, Marchiori M. Efficient behavior of small-world networks. *Phys Rev Lett.* 2001;87(19):198701.

5.	Rubinov M, Sporns O. Complex network measures of brain connectivity: uses and interpretations. *Neuroimage.* 2010;52(3):1059–1069.

6.	van den Heuvel MP, Sporns O. Rich-club organization of the human connectome. *J Neurosci.* 2011;31(44):15775–15786.

7.	Vos de Wael R, Benkarim O, Paquola C, et al. BrainSpace: a toolbox for the analysis of macroscale gradients in neuroimaging and connectomics datasets. *Commun Biol.* 2020;3(1):103.

8.	Power JD, Barnes KA, Snyder AZ, Schlaggar BL, Petersen SE. Spurious but systematic correlations in functional connectivity MRI networks arise from subject motion. *Neuroimage.* 2012;59(3):2142–2154.

9.	Fan L, Li H, Zhuo J, et al. The Human Brainnetome Atlas: A New Brain Atlas Based on Connectional Architecture. *Cereb Cortex.* 2016;26(8):3508–3526.

10.	Diedrichsen J. A spatially unbiased atlas template of the human cerebellum. *Neuroimage.* 2006;33(1):127–138.

11.	Diedrichsen J, Balsters JH, Flavell J, Cussans E, Ramnani N. A probabilistic MR atlas of the human cerebellum. *Neuroimage.* 2009;46(1):39–46.

12.	Diedrichsen J, Maderwald S, Kuper M, et al. Imaging the deep cerebellar nuclei: a probabilistic atlas and normalization procedure. *Neuroimage.* 2011;54(3):1786–1794.

13.	Schaefer A, Kong R, Gordon EM, et al. Local-Global Parcellation of the Human Cerebral Cortex from Intrinsic Functional Connectivity MRI. *Cereb Cortex.* 2018;28(9):3095–3114.

14.	Tian Y, Margulies DS, Breakspear M, Zalesky A. Topographic organization of the human subcortex unveiled with functional connectivity gradients. *Nat Neurosci.* 2020;23(11):1421–1432.

15.	Austin PC. An Introduction to Propensity Score Methods for Reducing the Effects of Confounding in Observational Studies. *Multivariate Behav Res.* 2011;46(3):399–424.
