# Supplementary material for: Disrupted Functional Network Organization in Crohn's Disease: Interoceptive Alterations Linked to Anxiety
Source: CNS Neurosci Ther. 2026 Aug 30;32(9):e71113. doi: 10.1002/cns.71113 (PMC13526404; doi:10.1002/cns.71113)
Supplement: Supplementary file 2 [file CNS-32-e71113-s003.docx]

| **Supplementary Table 1. Hub regions in CD patients and HCs** | | | | | | | |
| --- | --- | --- | --- | --- | --- | --- | --- |
| **Hub Regions** | **MNI Centroid** | | | **Hub Regions** | **MNI Centroid** | | |
|  | **X** | **Y** | **Z** |  | **X** | **Y** | **Z** |
| **common hub regions in Parcellation 1** | | | | | | | |
| Superior Frontal Gyrus.L | -6 | -5 | 58 | Inferior Parietal Lobule.R | 53 | -54 | 25 |
| Superior Frontal Gyrus.R | 7 | -4 | 60 | Inferior Parietal Lobule.L | -53 | -31 | 23 |
| Superior Frontal Gyrus.R | 6 | 38 | 35 | Inferior Parietal Lobule.R | 55 | -26 | 26 |
| Inferior Frontal Gyrus.R | 42 | 22 | 3 | Postcentral Gyrus.L | -56 | -14 | 16 |
| Inferior Frontal Gyrus.L | -52 | 13 | 6 | Postcentral Gyrus.R | 56 | -10 | 15 |
| Orbital Gyrus.R | 42 | 31 | -9 | Postcentral Gyrus.R | 48 | -24 | 48 |
| Precentral Gyrus.L | -32 | -9 | 58 | Postcentral Gyrus.L | -21 | -35 | 68 |
| Precentral Gyrus.R | 33 | -7 | 57 | Insular Gyrus.L | -36 | -20 | 10 |
| Precentral Gyrus.L | -52 | 0 | 8 | Insular Gyrus.L | -34 | 18 | 1 |
| Precentral Gyrus.R | 54 | 4 | 9 | Insular Gyrus.L | -38 | -4 | -9 |
| Precentral Gyrus.R | 51 | 7 | 30 | Insular Gyrus.R | 39 | -2 | -9 |
| Paracentral Lobule.R | 10 | -34 | 54 | Insular Gyrus.R | 38 | 5 | 5 |
| Superior Temporal Gyrus.R | 54 | -24 | 11 | Cingulate Gyrus.L | -5 | 7 | 37 |
| Superior Temporal Gyrus.L | -50 | -11 | 1 | Cingulate Gyrus.R | 4 | 6 | 38 |
| Superior Temporal Gyrus.R | 51 | -4 | -1 | Cingulate Gyrus.L | -7 | -23 | 41 |
| Middle Temporal Gyrus.L | -58 | -20 | -9 | Cingulate Gyrus.R | 6 | -20 | 40 |
| Fusiform Gyrus.L | -42 | -51 | -17 | Basal Ganglia.R | 29 | -3 | 1 |
| **different hub region in CD in Parcellation 1** | | | | **different hub region in HC in Parcellation 1** | | | |
| Superior Frontal Gyrus.R | 7 | 16 | 54 | Paracentral Lobule.R | 5 | -21 | 61 |
| Superior Frontal Gyrus.R | 20 | 4 | 64 | Superior Temporal Gyrus.L | -54 | -32 | 12 |
| Inferior Frontal Gyrus.L | -39 | 23 | 4 | Postcentral Gyrus.R | 20 | -33 | 69 |
| Inferior Frontal Gyrus.R | 54 | 14 | 11 | Insular Gyrus.R | 37 | -18 | 8 |
| Orbital Gyrus.L | -41 | 32 | -9 | Insular Gyrus.R | 36 | 18 | 1 |
| Precentral Gyrus.L | -49 | 5 | 30 | Insular Gyrus.R | 39 | -7 | 8 |
| Paracentral Lobule.L | -8 | -38 | 58 | Basal Ganglia.L | -28 | -5 | 2 |
| Middle Temporal Gyrus.L | -59 | -58 | 4 | Thalamus.R | 7 | -11 | 6 |
| Middle Temporal Gyrus.R | 58 | -16 | -10 | Thalamus.L | -16 | -24 | 6 |
| Inferior Temporal Gyrus.L | -55 | -60 | -6 | Thalamus.L | -11 | -14 | 2 |
| Inferior Parietal Lobule.L | -47 | -65 | 26 | Thalamus.R | 13 | -17 | 7 |
| Insular Gyrus.L | -38 | 5 | 5 |  |  |  |  |
| Lateral Occipital Cortex.L | -46 | -74 | 3 |  |  |  |  |
| The table lists hub regions that were common to both groups, as well as hubs uniquely identified in CD patients or in HCs. Hub regions were defined whose degree ranked within the top 16% of all nodes in the group-level functional network. For each hub region, the anatomical label and centroid coordinates are provided. All coordinates are reported in MNI space. | | | | | | | |

| **Supplementary Table 2. Significant differences in Rich-club properties between CD and HCs** | | | | | | | | | |
| --- | --- | --- | --- | --- | --- | --- | --- | --- | --- |
| **CD vs HCs** | **Rich-club coefficient** | | **Normalized Rich-club coefficient** | | **Rich-club edges Erc** | | **Rich-club nodes Nrc** | |  |
| **k level** | **t value** | **p value** | **t value** | **p value** | **t value** | **p value** | **t value** | **p value** | |
| 1 | 1.82 | 0.071 | **1.98** | **0.049^*^** | 2.50 | 0.012 | **2.48** | **0.014^*^** | |
| 2 | 0.87 | 0.383 | 0.26 | 0.792 | 0.49 | 0.623 | **2.04** | **0.043^*^** | |
| 3 | 0.44 | 0.664 | 0.67 | 0.507 | 1.12 | 0.265 | **2.00** | **0.048^*^** | |
| 4 | 0.52 | 0.605 | 0.86 | 0.390 | 0.84 | 0.401 | 1.81 | 0.073 | |
| 5 | 0.36 | 0.719 | 1.16 | 0.248 | 0.94 | 0.349 | 1.76 | 0.080 | |
| 6 | 0.65 | 0.519 | 0.19 | 0.853 | 1.01 | 0.316 | 1.70 | 0.091 | |
| 7 | 0.05 | 0.958 | 0.18 | 0.857 | 1.62 | 0.106 | 1.96 | 0.052 | |
| 8 | -0.15 | 0.878 | 0.49 | 0.626 | 1.46 | 0.145 | 1.84 | 0.068 | |
| 9 | 0.49 | 0.627 | 1.05 | 0.295 | 1.12 | 0.263 | 1.73 | 0.085 | |
| 10 | -0.06 | 0.950 | 0.45 | 0.651 | 1.29 | 0.199 | 1.84 | 0.067 | |
| 11 | -0.28 | 0.779 | 0.74 | 0.462 | 1.11 | 0.271 | 1.82 | 0.071 | |
| 12 | 0.02 | 0.986 | 0.71 | 0.481 | 0.65 | 0.515 | 1.69 | 0.092 | |
| 13 | 0.24 | 0.810 | 0.81 | 0.418 | 0.82 | 0.413 | 1.83 | 0.069 | |
| 14 | 0.45 | 0.653 | 0.62 | 0.538 | 1.06 | 0.293 | 1.94 | 0.054 | |
| 15 | 0.04 | 0.965 | 0.41 | 0.684 | 1.22 | 0.224 | **2.09** | **0.039^*^** | |
| 16 | -0.52 | 0.605 | 0.57 | 0.571 | 1.03 | 0.304 | **2.07** | **0.040^*^** | |
| 17 | -0.87 | 0.385 | 0.56 | 0.580 | 0.60 | 0.551 | 1.94 | 0.055 | |
| 18 | -1.25 | 0.212 | 0.71 | 0.479 | 0.26 | 0.796 | 1.89 | 0.061 | |
| 19 | -1.43 | 0.156 | 0.60 | 0.547 | -0.22 | 0.830 | 1.73 | 0.085 | |
| 20 | -1.30 | 0.196 | 0.68 | 0.497 | -0.49 | 0.626 | 1.72 | 0.087 | |
| 21 | -1.35 | 0.179 | 0.40 | 0.691 | -0.21 | 0.837 | 1.88 | 0.062 | |
| 22 | -1.34 | 0.181 | 0.39 | 0.695 | -0.13 | 0.899 | **2.06** | **0.041^*^** | |
| 23 | -1.27 | 0.206 | 0.31 | 0.760 | -0.20 | 0.840 | **2.13** | **0.035^*^** | |
| 24 | -1.57 | 0.119 | 0.14 | 0.893 | -0.24 | 0.812 | **2.30** | **0.023^*^** | |
| 25 | -1.78 | 0.077 | 0.24 | 0.814 | -0.76 | 0.450 | **2.22** | **0.028^*^** | |
| 26 | -1.70 | 0.090 | 0.26 | 0.799 | -1.19 | 0.235 | **2.04** | **0.043^*^** | |
| 27 | -1.85 | 0.066 | 0.39 | 0.699 | -1.06 | 0.293 | **2.18** | **0.031^*^** | |
| 28 | -1.95 | 0.052 | 0.34 | 0.737 | -1.32 | 0.188 | 1.94 | 0.054 | |
| 29 | **-2.30** | **0.023^*^** | 0.70 | 0.487 | -1.45 | 0.148 | 1.91 | 0.059 | |
| 30 | **-2.22** | **0.028^*^** | 0.81 | 0.418 | **-2.10** | **0.037^*^** | 1.19 | 0.234 | |
| 31 | **-2.40** | **0.018^*^** | 0.80 | 0.424 | **-2.00** | **0.047^*^** | 1.09 | 0.278 | |
| 32 | **-2.44** | **0.016^*^** | 0.98 | 0.331 | **-2.25** | **0.026^*^** | 0.67 | 0.505 | |
| 33 | **-2.45** | **0.015^*^** | 1.01 | 0.313 | **-2.29** | **0.023^*^** | 0.16 | 0.872 | |
| 34 | **-2.50** | **0.013^*^** | 0.91 | 0.362 | **-2.39** | **0.018^*^** | -0.20 | 0.842 | |
| 35 | **-2.50** | **0.013^*^** | 0.96 | 0.338 | **-2.40** | **0.018^*^** | -0.63 | 0.529 | |
| 36 | **-2.36** | **0.019^*^** | 0.87 | 0.386 | **-2.42** | **0.017^*^** | -1.16 | 0.249 | |
| 37 | **-2.41** | **0.017^*^** | 1.16 | 0.249 | **-2.17** | **0.032^*^** | -0.99 | 0.323 | |
| 38 | **-2.49** | **0.014^*^** | 1.18 | 0.241 | **-2.32** | **0.022^*^** | -1.69 | 0.094 | |
| 39 | **-2.44** | **0.016^*^** | 1.24 | 0.216 | **-2.43** | **0.016^*^** | **-2.14** | **0.034^*^** | |
| 40 | **-2.43** | **0.016^*^** | 1.17 | 0.242 | **-2.35** | **0.020^*^** | **-2.17** | **0.032^*^** | |
| Differences in four Rich-club properties between CD patients and HCs at a series of k levels. A series of two-sample t tests were performed, and * means statistically significant p < 0.05 without any multiple comparison corrections. Erc: Number of edges in the rich club; Nrc: Number of nodes in the rich club | | | | | | | | | |
|  |  |  |  |  |  |  |  |  |  |

| **Supplementary Table 3. Significant differences in nodal DC and G1 between CD and HCs** | | | | | | |
| --- | --- | --- | --- | --- | --- | --- |
| **CD vs HCs** | **Centroid** | | | **region in AAL3** | **t value** | **FDR-q value** |
|  | **X** | **Y** | **Z** |  |  |  |
| **Node degree in Parcellation 1** |  |  |  |  |  |  |
| Superior Frontal Gyrus.L | -6 | -5 | 58 | Supp_Motor_Area_L | -3.10 | **0.032^*^** |
| Superior Temporal Gyrus.L | -50 | -11 | 1 | Temporal_Sup_L | -3.36 | **0.023^*^** |
| Superior Temporal Gyrus.R | 51 | -4 | -1 | Insula_R | -3.17 | **0.032^*^** |
| Inferior Temporal Gyrus.R | 55 | -11 | -32 | Temporal_Inf_R | -2.90 | **0.048^*^** |
| Fusiform Gyrus.L | -33 | -16 | -32 | Fusiform_L | -3.08 | **0.032^*^** |
| Precuneus.L | -12 | -67 | 25 | Cuneus_L | -3.08 | **0.032^*^** |
| Precuneus.R | 16 | -64 | 25 | Precuneus_R | -3.03 | **0.034^*^** |
| Postcentral Gyrus.L | -56 | -14 | 16 | Postcentral_L | -3.29 | **0.027^*^** |
| Insular Gyrus.L | -36 | -20 | 10 | Insula_L | -3.96 | **0.009^**^** |
| Insular Gyrus.R | 37 | -18 | 8 | Insula_R | -3.94 | **0.009^**^** |
| Insular Gyrus.R | 39 | -2 | -9 | - | -3.78 | **0.011^*^** |
| Insular Gyrus.L | -38 | -8 | 8 | Insula_L | -3.26 | **0.027^*^** |
| Insular Gyrus.R | 39 | -7 | 8 | - | -3.16 | **0.032^*^** |
| MedioVentral Occipital Cortex.L | -13 | -68 | 12 | Calcarine_L | -3.41 | **0.023^*^** |
| MedioVentral Occipital Cortex.R | 15 | -63 | 12 | Calcarine_R | -3.11 | **0.032^*^** |
| Putamen.R | 29 | -3 | 1 | Putamen_R | -3.49 | **0.023^*^** |
| Thalamus.L | -7 | -12 | 5 | Thal_VL_L | -3.39 | **0.023^*^** |
| Thalamus.L | -18 | -13 | 3 | Thal_VL_L | -3.08 | **0.032^*^** |
| Thalamus.R | 12 | -14 | 1 | Thal_VL_R | -3.37 | **0.023^*^** |
| Thalamus.L | -18 | -23 | 4 | Thal_VPL_L | -4.42 | **0.005^**^** |
| Thalamus.R | 18 | -22 | 3 | Thal_VPL_R | -3.95 | **0.009^**^** |
| Thalamus.L | -16 | -24 | 6 | Thal_PuA_L | -3.43 | **0.023^*^** |
| Thalamus.R | 15 | -25 | 6 | Thal_PuM_R | -3.03 | **0.034^*^** |
| Thalamus.L | -11 | -14 | 2 | Thal_VL_L | -3.14 | **0.032^*^** |
| Thalamus.R | 13 | -16 | 7 | Thal_VL_R | -3.76 | **0.011^*^** |
| **Gradient scores in Parcellation 2** | | | | | | |
| lVisCent_ExStr_9 | -39 | -84 | 14 | Occipital_Mid_L | -3.32 | **0.041^*^** |
| lVisCent_ExStr_11 | -25 | -85 | 21 | Occipital_Mid_L | -3.43 | **0.038^*^** |
| lSomMotB_Ins_1 | -36 | -24 | 10 | Heschl_L  Insula_L | 3.63 | **0.032^*^** |
| lSomMotB_S2_2 | -36 | -26 | 19 | Insula_L | 3.26 | **0.041^*^** |
| lSomMotB_S2_3 | -49 | -13 | 14 | Rolandic_Oper_L | 3.52 | **0.032^*^** |
| lSomMotB_S2_5 | -48 | -24 | 18 | Rolandic_Oper_L | 3.84 | **0.018^*^** |
| lDorsAttnA_SPL_5 | -36 | -52 | 56 | Parietal_Inf_L | -3.25 | **0.041^*^** |
| lDorsAttnB_PostC_3 | -55 | -32 | 45 | Parietal_Inf_L  Postcentral_L | -4.47 | **0.007^**^** |
| lDorsAttnB_PostC_4 | -46 | -29 | 44 | Parietal_Inf_L | -4.22 | **0.007^**^** |
| lDorsAttnB_FEF_2 | -25 | -1 | 55 | Frontal_Mid_2_L | -3.54 | **0.032^*^** |
| lContA_IPS_4 | -45 | -41 | 47 | Parietal_Inf_L | -3.89 | **0.018^*^** |
| lContA_IPS_5 | -33 | -46 | 41 | Parietal_Inf_L | -3.30 | **0.041^*^** |
| rSomMotB_S2_2 | 35 | -21 | 14 | Insula_R | 3.29 | **0.041^*^** |
| rDorsAttnA_ParOcc_3 | 36 | -79 | 24 | Occipital_Mid_R | -4.27 | **0.007^**^** |
| rDorsAttnA_SPL_7 | 27 | -58 | 61 | Parietal_Sup_R | -3.51 | **0.032^*^** |
| All results were corrected using FDR at q<0.05. ^*^ indicates results significant at q<0.05, and ^**^ indicates results significant q<0.01. **Abbreviations:** Frontal_Mid: Middle Frontal Gyrus; Occipital_Mid: Middle Occipital Gyrus; Parietal_Inf: Inferior Parietal Gyrus; Parietal_Sup: Superior Parietal Gyrus; Rolandic_Oper: Rolandic Operculum; Supp_Motor_Area: Supplementary Motor Area; Temporal_Sup: Superior Temporal Gyrus; Temporal_Inf: Inferior Temporal Gyrus; Thal_VL: Ventral Lateral Nucleus; Thal_VPL: Ventral Posterolateral Nucleus; Thal_PuA_L: Pulvinar Anterior Nucleus; VisCent_ExStr: Central Visual, Extrastriate Cortex; SomMotB_Ins: Somatomotor B, Insula; SomMotB_S2: Somatomotor B, Secondary Somatosensory Cortex; DorsAttnA_ParOcc: Dorsal Attention A, Parietal Occipital; DorsAttnA_SPL: Dorsal Attention A, Superior Parietal Lobule; DorsAttnB_PostC: Dorsal Attention B, Postcentral; DorsAttnB_FEF: Dorsal Attention B, Frontal Eye Fields; ContA_IPS: Control A, Intraparietal Sulcus; | | | | | | |

| **Supplementary Table 4. Significant differences in distribution of gradient scores in Sub-networks between CD patients and HCs** | | |
| --- | --- | --- |
| **sub-network** | **Z value** | **FDR-q value** |
| DMN | -0.54 | 0.676 |
| TPN | 0.09 | 0.930 |
| ECN | -2.65 | **0.020^*^** |
| Limbic | -0.96 | 0.485 |
| Cerebellum | 1.77 | 0.128 |
| Subcortical | 2.73 | **0.020^*^** |
| VN | -1.98 | 0.095 |
| DAN | -4.07 | **<0.001^**^** |
| VAN | 0.51 | 0.676 |
| SMN | 2.75 | **0.020^*^** |
| Distribution of differences in gradients score in sub-networks using the nonparametric Mann-Whitney U tests between CD patients and HCs, and * means statistically significant FDR-q < 0.05. DMN: default mode network; TPN: temporal parietal network; ECN: executive control network; Limbic: limbic system; VN: visual network; DAN: dorsal attention network; VAN: ventral attention network; SMN: somatomotor network; | | |
|  |  |  |
|  |  |  |
|  |  |  |

| **Supplementary Table 5. Post-hoc linear regression and interaction analyses of DC values in CD patients** | | | | | |
| --- | --- | --- | --- | --- | --- |
| **Brain regions** | | **β value（95% CI）** | | **t value** | **p value** |
| **Association of DC with CDAI** | | | | | |
| Putamen.L | CDAI | **-0.51 (-0.69 to -0.33)** | | **-5.75** | **<0.001^**^** |
| Putamen.R | CDAI | **-0.49 (-0.67 to -0.30)** | | **-5.26** | **<0.001^**^** |
| **Interaction of DC, CDAI, and HAD-A** | | | | | |
| Extending from Parietal_Inf_L to Postcentral_L and Precentral_L | CDAI | 0.14 | (-0.04 to 0.33) | 1.53 | 0.130 |
|  | HAD-A | -0.07 | (-0.25 to 0.11) | -0.79 | 0.433 |
|  | CDAI*HAD-A | **0.41** | **( 0.22 to 0.60)** | **4.38** | **<0.001^**^** |
| Data are presented as β (95% CI). Linear models were adjusted for age, sex, education duration, and mean framewise displacement. ** indicates results significant p<0.001. Abbreviations: DC, degree centrality; CDAI, Crohn's Disease Activity Index; HAD-A, Hospital Anxiety and Depression Scale–Anxiety subscale; CI, confidence interval. | | | | | |

| **Supplementary Table 6. Significant effective connectivity associated with anxiety (HAD-A) in CD patients** | | |
| --- | --- | --- |
| **Effective connectivity** | **Ep** | **Pp** |
| **Extrinsic connections** | | |
| From Ins.L to PoG&IPL.L | 0.012 | **> 0.99** |
| From Ins.L to Put.L | 0.010 | **> 0.99** |
| From Ins.L to Tha.L | -0.014 | **> 0.99** |
| From Ins.L to Tha.R | -0.010 | **> 0.99** |
| From Ins.R to Ins.L | -0.040 | **> 0.99** |
| From Ins.R to PoG&IPL.R | 0.008 | **> 0.99** |
| From Ins.R to Tha.L | 0.016 | **> 0.99** |
| From Ins.R to Tha.R | 0.011 | **> 0.99** |
| From PoG&IPL.L to Put.L | 0.016 | **> 0.99** |
| From PoG&IPL.L to Put.R | 0.011 | **> 0.99** |
| From PoG&IPL.L to Tha.L | 0.012 | **> 0.99** |
| From PoG&IPL.L to Tha.R | -0.010 | **> 0.99** |
| From PoG&IPL.R to Put.L | -0.009 | **> 0.99** |
| From Put.L to Ins.L | -0.015 | **> 0.99** |
| From Put.R to Ins.L | -0.013 | **> 0.99** |
| From Put.R to Put.L | -0.011 | **> 0.99** |
| From Tha.L to Ins.L | -0.024 | **> 0.99** |
| From Tha.L to PoG&IPL.L | -0.010 | **> 0.99** |
| From Tha.L to Put.L | 0.015 | **> 0.99** |
| From Tha.L to Tha.R | -0.009 | **> 0.99** |
| From Tha.R to Ins.L | 0.030 | **> 0.99** |
| From Tha.R to PoG&IPL.L | 0.010 | **> 0.99** |
| From Tha.R to Tha.L | 0.009 | **> 0.99** |
| **Self-connections** | | |
| From Ins.L to Ins.L | -0.060 | **> 0.99** |
| From Ins.R to Ins.R | -0.011 | **> 0.99** |
| From PoG&IPL.L to PoG&IPL.L | 0.016 | **> 0.99** |
| From PoG&IPL.R to PoG&IPL.R | 0.016 | **> 0.99** |
| From Tha.L to Tha.L | 0.027 | **> 0.99** |
| Significant associations between effective connectivity and anxiety (HAD-A) in CD patients. Ep means expected parameter and Pp means posterior probability of the effect. Ins: insula; Put: putamen; Tha: thalamus; PoG: postcentral gyrus; IPL: inferior parietal lobule. | | |
|  |  |  |

| **Supplementary Table 7. Significant differences in global properties and rich club between CD patients and HCs based on Parcellation 2 (final analytic sample, n=161)** | | |
| --- | --- | --- |
| **CD vs HCs** | **t value** | **FDR-q value** |
|  |  |  |
| **Global properties measure the overall structural characteristics of a functional network** | | |
| Shortest Path Length | 4.41 | **<0.001^**^** |
| Clustering Coefficient | -4.11 | **<0.001^**^** |
| Network efficiency | -4.20 | **<0.001^**^** |
| Network local efficiency | -3.98 | **<0.001^**^** |
| **Rich-club indicates a network's subset of highly interconnected nodes** | | |
| Rich-club connections | -3.09 | **0.003^**^** |
| Feeder connections | -3.30 | **0.002^**^** |
| Local connections | -3.61 | **0.002^**^** |
| Distance among hub regions | 2.06 | **0.041^*^** |
| All results were corrected using FDR at q<0.05. * q<0.05; ** q<0.01. Rich-club connections refer to connections between hub regions (top 16% ranked by degree); feeder connections refer to connections between hub and non-hub regions; local connections refer to connections between non-hub regions. | | |

| **Supplementary Table 8. Significant differences in nodal DC between CD and HCs based on Parcellation 2 (final analytic sample, n=161)** | | | | | | |
| --- | --- | --- | --- | --- | --- | --- |
| **CD vs HCs** | **Centroid** | | | **region in AAL3** | **t value** | **FDR-q value** |
|  | **X** | **Y** | **Z** |  |  |  |
| **Node degree in Parcellation 2** | | | | | | |
| lVisPeri_ExStrInf_5 | -14 | -57 | 1 | Lingual_L | -3.80 | **0.013^*^** |
| lVisPeri_StriCal_2 | -7 | -74 | 9 | Calcarine_L | -3.32 | **0.023^*^** |
| lVisPeri_ExStrSup_1 | -19 | -65 | 7 | Calcarine_L | -3.17 | **0.031^*^** |
| lVisPeri_ExStrSup_3 | -12 | -71 | 20 | Calcarine_L | -3.42 | **0.020^*^** |
| lSomMotA_6 | -9 | -38 | 54 | Cingulate_Mid_L | -2.98 | **0.039^*^** |
| lSomMotA_13 | -19 | -24 | 67 | Precentral_L | -3.31 | **0.023^*^** |
| lSomMotB_Aud_1 | -50 | -9 | 0 | Temporal_Sup_L | -2.89 | **0.046^*^** |
| lSomMotB_Ins_1 | -36 | -24 | 10 | Heschl_L | -3.54 | **0.016^*^** |
| lSomMotB_S2_2 | -36 | -26 | 19 | Insula_L | -4.07 | **0.013^*^** |
| lSomMotB_S2_3 | -49 | -13 | 14 | Rolandic_Oper_L | -3.62 | **0.014^*^** |
| lSomMotB_S2_4 | -60 | -2 | 11 | Rolandic_Oper_L | -3.50 | **0.016^*^** |
| lSomMotB_S2_5 | -48 | -24 | 18 | Rolandic_Oper_L | -3.14 | **0.032^*^** |
| lSomMotB_Cent_1 | -59 | -2 | 23 | Postcentral_L | -3.054 | **0.035^*^** |
| lSomMotB_Cent_4 | -51 | -7 | 43 | Postcentral_L | -2.934 | **0.043^*^** |
| lSalVentAttnA_Ins_1 | -39 | 2 | -4 | Insula_L | -2.90 | **0.046^*^** |
| lSalVentAttnA_Ins_2 | -40 | -15 | -2 | Insula_L | -4.15 | **0.013^*^** |
| lSalVentAttnA_FrMed_2 | -5 | 9 | 48 | Supp_Motor_Area_L | -3.31 | **0.023^*^** |
| lSalVentAttnA_FrMed_3 | -8 | -3 | 71 | Supp_Motor_Area_L | -3.60 | **0.014^*^** |
| lLimbicA_TempPole_1 | -37 | -5 | -42 | Temporal_Inf_L | -2.99 | **0.039^*^** |
| lContC_pCun_1 | -10 | -70 | 32 | Precuneus_L | -3.98 | **0.013^*^** |
| rVisPeri_StriCal_1 | 9 | -74 | 9 | Calcarine_R | -3.69 | **0.013^*^** |
| rVisPeri_ExStrSup_1 | 16 | -66 | 19 | Cuneus_R | -3.72 | **0.013^*^** |
| rSomMotB_Aud_2 | 53 | -14 | 6 | Heschl_R | -3.09 | **0.033^*^** |
| rSomMotB_S2_2 | 35 | -21 | 14 | Insula_R | -3.52 | **0.016^*^** |
| rSalVentAttnA_Ins_1 | 40 | 5 | -15 | Insula_R | -3.02 | **0.037^*^** |
| rSalVentAttnA_Ins_3 | 40 | -10 | -4 | Insula_R | -3.33 | **0.023^*^** |
| rSalVentAttnA_ParMed_4 | 11 | -32 | 50 | Paracentral_Lobule_R | -3.11 | **0.032^*^** |
| rSalVentAttnB_Ins_2 | 37 | 23 | 5 | Insula_R | -3.10 | **0.032^*^** |
| rContA_PFCl_5 | 39 | 11 | 34 | Frontal_Inf_Oper_R | -3.68 | **0.013^*^** |
| rContB_PFClv_1 | 35 | 38 | -13 | Frontal_Inf_Orb_2_R | -3.02 | **0.037^*^** |
| rContC_pCun_1 | 17 | -63 | 28 | Precuneus_R | -3.08 | **0.033^*^** |
| rContC_pCun_2 | 13 | -71 | 39 | Precuneus_R | -3.16 | **0.031^*^** |
| rTHA-VAip | 8 | -14 | 2 | Thal_MDl_R | -3.77 | **0.013^*^** |
| rTHA-DPl | 17 | -30 | 0 | Thal_PuI_R | -3.25 | **0.027^*^** |
| rTHA-VPm | 12 | -21 | 1 | Thal_IL_R | -3.11 | **0.032^*^** |
| rTHA-DAl | 14 | -19 | 13 | Thal_VPL_R | -3.84 | **0.013^*^** |
| rPUT-VP | 28 | -9 | -1 | Pallidum_R | -3.18 | **0.030^*^** |
| rPUT-DP | 26 | 0 | 6 | Putamen_R | -3.23 | **0.027^*^** |
| lTHA-VAip | -8 | -14 | 2 | Thal_MDl_L | -3.19 | **0.030^*^** |
| lTHA-VPl | -18 | -23 | 5 | Thal_VPL_L | -3.67 | **0.013^*^** |
| lTHA-DAl | -14 | -19 | 13 | Thal_VPL_L | -4.02 | **0.013^*^** |
| lCerebellum_VIIb | -28 | -65 | -51 | Cerebellum_8_L | -2.87 | **0.049^*^** |
| rCerebellum_VIIb | 28 | -65 | -50 | Cerebellum_8_R | -3.43 | **0.020^*^** |
| lCerebellum_VIIIa | -24 | -58 | -53 | Cerebellum_8_L | -2.98 | **0.039^*^** |
| rCerebellum_VIIIa | 25 | -58 | -53 | Cerebellum_8_R | -3.90 | **0.013^*^** |
| lCerebellum_VIIIb | -17 | -51 | -55 | Cerebellum_9_L | -3.35 | **0.023^*^** |
| Cerebellum_Vemis_X | 1 | -49 | -36 | Vermis_10 | -3.72 | **0.013^*^** |
| rCerebellum_X | 21 | -38 | -45 | Cerebellum_10_R | -2.96 | **0.040^*^** |
| All results were corrected using FDR at q<0.05. ^*^ q<0.05, ^**^ q<0.01. **Abbreviations:** VisPeri_ExStrInf: Visual Peripheral, Extra-striate Inferior; VisPeri_StriCal: Visual Peripheral, Striate Calcarine; VisPeri_ExStrSup: Visual Peripheral, Extra-striate Superior; SomMotA: Somatomotor A; SomMotB_Aud: Somatomotor B, Auditory; SomMotB_Ins: Somatomotor B, Insula; SomMotB_S2: Somatomotor B, Secondary Somatosensory Cortex; SomMotB_Cent: Somatomotor B, Central; SalVentAttnA_Ins: Salience/Ventral Attention A, Insula; SalVentAttnA_ParMed: Salience/Ventral Attention A, Parietal Medial; SalVentAttnA_FrMed: Salience/Ventral Attention A, Frontal Medial; SalVentAttnB_Ins: Salience/Ventral Attention B, Insula; LimbicA_TempPole: Limibic A, Temporal Pole; ContA_PFCl: Control A, Lateral Prefrontal Cortex; ContB_PFClv: Lateral Ventral Prefrontal Cortex; ContC_pCun: Control C, Precuneus; THA-VAip; thalamus posterior division; THA-DPl; thalamus lateral dorsoposterior; THA-VPl; thalamus lateral ventroposterior; THA-VPm; thalamus medial ventroposterior; THA-DAl; thalamus lateral dorsoanterior; PUT-VP; putamen ventroposterior; PUT-DP; putamen dorsoposterior; | | | | | | |

| **Supplementary Table 9. Demographic and clinical characteristics of CD patients and HCs in the demographically matched subsample (n=122)** | | | |
| --- | --- | --- | --- |
|  | **CD (n=61)** | **HCs (n=61)** | **p value** |
| **Sex (male/female)** | 42/19 | 37/24 | 0.309 |
| **Age (y)** | 31.7±8.4 | 33.0±8.6 | 0.408 |
| **Education (y)** | 17.4±3.1 | 17.9±3.1 | 0.431 |
| **BMI** | 20.1±3.1 | - | - |
| **Duration of CD (months)** | 52.7±46.2 | - | - |
| **VAS maximum abdominal pain** | 2.5±2.1 | - | - |
| **VAS average abdominal pain** | 1.8±1.6 | - | - |
| **CDAI score** | 126.9±73.1 | - | - |
| **IBDQ** | 171.7±26.8 | - | - |
| **HADS-A** | 5.8±3.4 | - | - |
| **HADS-D** | 4.6±3.3 | - | - |
| * indicates statistical significance at p<0.05, and ** indicates statistical significance at p<0.01. Abbreviations: BMI: Body Mass Index; VAS: Visual Analogue Scale; CDAI: Crohn's Disease Activity Index; IBDQ: Inflammatory Bowel Disease Questionnaire; HAD-A/HAD-D: Hospital Anxiety and Depression Scale. | | | |

| **Supplementary Table 10. Sensitivity analyses of global properties and rich-club organization: sex-stratified subsamples (males, n = 113; females, n = 48) and demographically matched subsample (n = 122), based on Parcellation 1.** | | | | | | |
| --- | --- | --- | --- | --- | --- | --- |
| **CD vs HCs** | **Males (n=113)** | | **Females (n=48)** | | **Matched (n=122)** | |
|  | **t value** | **FDR-q value** | **t value** | **FDR-q value** | **t value** | **FDR-q value** |
| Shortest Path Length | 2.04 | 0.098 | 1.23 | 0.226 | 2.79 | **0.011^*^** |
| Clustering Coefficient | -1.10 | 0.273 | -1.23 | 0.226 | -2.20 | **0.030^*^** |
| Network efficiency | -1.99 | 0.098 | -1.23 | 0.226 | -2.72 | **0.011^*^** |
| Network local efficiency | -1.79 | 0.101 | -1.49 | 0.226 | -2.82 | **0.011^*^** |
| Rich-club connections | -1.24 | 0.217 | -1.14 | 0.500 | -2.54 | **0.023^*^** |
| Feeder connections | -1.78 | 0.156 | -0.69 | 0.500 | -2.30 | **0.023^*^** |
| Local connections | -2.06 | 0.156 | -0.68 | 0.500 | -2.32 | **0.023^*^** |
| Distance among hub regions | 1.45 | 0.200 | 1.42 | 0.500 | 2.52 | **0.023^*^** |
| All results were corrected using FDR at q<0.05. * q<0.05; ** q<0.01. Hub regions were defined as nodes ranked in the top 16% by degree. Rich-club connections refer to connections between hub regions; feeder connections refer to connections between hub and non-hub regions; local connections refer to connections between non-hub regions. | | | | | | |

| **Supplementary Table 11. Sensitivity analyses of AUC of DC and Gradient score 1: sex-stratified subsamples (males, n = 113; females, n = 48) and demographically matched subsample (n = 122).** | | | | | | |
| --- | --- | --- | --- | --- | --- | --- |
| **CD vs HCs** | **Post-Stratification** | | | | **Matched Subsample Analysis** | |
|  | **males** | | **females** | |  |  |
|  | **t value** | **FDR-q value** | **t value** | **FDR-q value** | **t value** | **FDR-q value** |
| **Significant group differences in DC (main, samples, n=161, Parcellation 1)** | | | | | | |
| Superior Frontal Gyrus.L | -1.73 | 0.106 | -1.48 | 0.252 | -2.72 | 0.094 |
| Superior Temporal Gyrus.L | **-2.15** | **0.049^*^** | -0.96 | 0.419 | -2.51 | 0.131 |
| Superior Temporal Gyrus.R | **-2.22** | **0.044^*^** | -0.37 | 0.715 | -2.33 | 0.152 |
| Inferior Temporal Gyrus.R | -1.55 | 0.142 | -2.91 | 0.071 | -2.33 | 0.152 |
| Fusiform Gyrus.L | **-2.30** | **0.044^*^** | -2.08 | 0.154 | **-3.20** | **0.049^*^** |
| Precuneus.L | **-2.45** | **0.039^*^** | -1.85 | 0.199 | -3.07 | 0.057 |
| Precuneus.R | **-2.23** | **0.044^*^** | -1.62 | 0.252 | -2.77 | 0.087 |
| Postcentral Gyrus.L | -1.71 | 0.106 | -1.47 | 0.252 | -2.66 | 0.100 |
| Insular Gyrus.L | -1.44 | 0.167 | -2.93 | 0.071 | **-3.28** | **0.049^*^** |
| Insular Gyrus.R | -2.11 | 0.052 | -1.76 | 0.213 | **-3.17** | **0.049^*^** |
| Insular Gyrus.R | **-2.59** | **0.030^*^** | -2.21 | 0.154 | **-3.40** | **0.049^*^** |
| Insular Gyrus.L | -0.79 | 0.431 | -2.40 | 0.142 | -2.59 | 0.109 |
| Insular Gyrus.R | -1.11 | 0.281 | -1.98 | 0.171 | -2.63 | 0.103 |
| MedioVentral Occipital Cortex.L | **-2.36** | **0.044^*^** | -1.52 | 0.252 | **-4.00** | **0.031^*^** |
| MedioVentral Occipital Cortex.R | -1.93 | 0.074 | -1.17 | 0.368 | **-3.52** | **0.049^*^** |
| Putamen.R | **-2.88** | **0.018^*^** | -1.24 | 0.349 | **-3.22** | **0.049^*^** |
| Thalamus.L | **-3.47** | **0.006^**^** | -0.94 | 0.419 | **-3.29** | **0.049^*^** |
| Thalamus.L | **-2.70** | **0.025^*^** | -0.79 | 0.493 | -2.86 | 0.082 |
| Thalamus.R | **-2.32** | **0.044^*^** | -2.36 | 0.142 | -2.71 | 0.094 |
| Thalamus.L | **-4.12** | **0.002^**^** | -1.12 | 0.374 | **-3.57** | **0.049^*^** |
| Thalamus.R | **-2.86** | **0.018^*^** | -2.10 | 0.154 | **-3.21** | **0.049^*^** |
| Thalamus.L | **-3.49** | **0.006^**^** | -0.47 | 0.669 | -2.81 | 0.082 |
| Thalamus.R | **-2.87** | **0.018^*^** | -0.51 | 0.668 | -2.45 | 0.131 |
| Thalamus.L | **-2.28** | **0.044^*^** | -1.46 | 0.252 | -2.47 | 0.131 |
| Thalamus.R | **-3.42** | **0.006^**^** | -1.07 | 0.381 | **-3.20** | **0.049^*^** |
| **Significant group differences in Gradient score 1 (main, samples, n=161, Parcellation 2)** | | | | | | |
| lVisCent_ExStr_9 | **-2.31** | **0.035****^*^** | -2.08 | 0.164 | -3.12 | 0.188 |
| lVisCent_ExStr_11 | -1.70 | 0.099 | **-3.20** | **0.039^*^** | -3.01 | 0.200 |
| lSomMotB_Ins_1 | **2.08** | **0.046^*^** | 2.11 | 0.164 | 2.66 | 0.300 |
| lSomMotB_S2_2 | **2.42** | **0.032^*^** | 0.83 | 0.477 | 2.00 | 0.449 |
| lSomMotB_S2_3 | **2.19** | **0.039^*^** | 0.93 | 0.458 | 1.91 | 0.461 |
| lSomMotB_S2_5 | **2.78** | **0.019^*^** | 1.60 | 0.251 | 2.39 | 0.315 |
| lDorsAttnA_SPL_5 | **-2.38** | **0.032^*^** | -1.19 | 0.362 | -1.99 | 0.449 |
| lDorsAttnB_PostC_3 | **-3.09** | **0.010^*^** | -1.45 | 0.263 | **-4.02** | **0.029^*^** |
| lDorsAttnB_PostC_4 | **-3.76** | **0.004^**^** | -1.44 | 0.263 | **-3.99** | **0.029^*^** |
| lDorsAttnB_FEF_2 | **-3.11** | **0.010^*^** | -0.91 | 0.458 | -3.30 | 0.125 |
| lContA_IPS_4 | **-2.42** | **0.032^*^** | -1.63 | 0.251 | -3.01 | 0.200 |
| lContA_IPS_5 | **-3.14** | **0.010^*^** | -0.25 | 0.805 | -2.44 | 0.303 |
| rSomMotB_S2_2 | **2.52** | **0.032^*^** | 0.68 | 0.536 | 1.82 | 0.461 |
| rDorsAttnA_ParOcc_3 | **-2.24** | **0.037^*^** | -1.78 | 0.244 | **-3.75** | **0.046^*^** |
| rDorsAttnA_SPL_7 | -1.49 | 0.138 | -2.15 | 0.164 | -2.85 | 0.259 |
| All results were corrected using FDR at q<0.05. ^*^ indicates results significant at q<0.05, and ^**^ indicates results significant q<0.01. **Abbreviations:** VisCent_ExStr: Central Visual, Extrastriate Cortex; SomMotB_Ins: Somatomotor B, Insula; SomMotB_S2: Somatomotor B, Secondary Somatosensory Cortex; DorsAttnA_ParOcc: Dorsal Attention A, Parietal Occipital; DorsAttnA_SPL: Dorsal Attention A, Superior Parietal Lobule; DorsAttnB_PostC: Dorsal Attention B, Postcentral; DorsAttnB_FEF: Dorsal Attention B, Frontal Eye Fields; ContA_IPS: Control A, Intraparietal Sulcus. | | | | | | |
