# Supplementary material for: Disrupted Functional Network Organization in Crohn's Disease: Interoceptive Alterations Linked to Anxiety
Source: CNS Neurosci Ther. 2026 Aug 30;32(9):e71113. doi: 10.1002/cns.71113 (PMC13526404; doi:10.1002/cns.71113)
Supplement: Supplementary file 3 [file CNS-32-e71113-s002.zip › cns71113-sup-0001-FigureS1-S4-TableS1-S11.docx]

**Supplementary Material Captions**

**Supplementary Figure 1. Rich-club across k-levels in CD patients and HCs**

(a) Rich-club coefficient (Φ) across degree thresholds (k). CD patients showed a significant reduction in Φ at higher k levels (k > 28). (b) Normalized rich-club coefficient (Φn) across degree thresholds, showing no significant group differences. (c) Total number of surviving edges across degree thresholds, with significantly fewer edges in CD patients at higher k levels (k > 32). (d) Total number of surviving nodes across degree thresholds, showing significantly higher node counts in CD patients at intermediate k ranges (k = 14–18 and 21–28). All results with p<0.05 (uncorrected for multiple comparisons) are indicated by *. Abbreviations: CD, Crohn's disease; HC, healthy control.

**Supplementary Figure 2. Group-averaged principal gradient and global/subnetwork gradient distributions in CD patients and HCs**

Average principal gradient of functional connectome in CD patients and HCs, showing a continuous axis from the sensory system (one extreme) to the transmodal default mode network (DMN) (other extreme). (b) Left panel: histogram of gradient scores across all brain regions for all participants, showing a unilateral compression pattern at the sensory end of the axis. Right panel: distributions of gradient scores within each subnetwork, with comparisons between CD patients and HCs. * indicates results of the Mann-Whitney U test surviving FDR correction at q<0.05. Abbreviations: DMN: default mode network; TPN: temporal parietal network; ECN: executive control network; Limbic: limbic system; VN: visual network; DAN: dorsal attention network; VAN: ventral attention network; SMN: somatomotor network;

**Supplementary Figure 3. Cross-sample replication of degree centrality group differences**

The figure shows the replication of degree centrality differences between CD patients and HCs across four independent samples. Sample 1 (Main Sample, n = 161) was used to identify the initial pattern of group differences. Samples 3 (Males, n = 113) and 4 (Females, n = 48) were used as post-hoc validation of the regions identified in Sample 1. Sample 2 (Matched Sample, n = 122) was analyzed across all brain regions, but only the results corresponding to the same regions identified in Sample 1 are displayed here for direct comparison (note that all significant regions from Sample 2 were retained). The direction of regional alterations remained consistent across all four samples. Significant group differences were identified using two-sample t-tests with FDR correction (p<0.05). Abbreviations: CD, Crohn's disease; HC, healthy control.

**Supplementary Figure 4. Cross-sample replication of Gradient 1 scores group differences**

The figure shows the replication of G1 differences between CD patients and HCs across four independent samples. Sample 1 (Main Sample, n = 161) was used to identify the initial pattern of group differences. Samples 3 (Males, n = 113) and 4 (Females, n = 48) were used as post-hoc validation of the regions identified in Sample 1. Sample 2 (Matched Sample, n = 122) was analyzed across all brain regions, but only the results corresponding to the same regions identified in Sample 1 are displayed here for direct comparison (note that all significant regions from Sample 2 were retained). The direction of regional alterations remained consistent across all four samples. Significant group differences were identified using two-sample t-tests with FDR correction (p<0.05). Abbreviations: CD, Crohn's disease; HC, healthy control.

**Supplementary Table 1.** Hub regions in CD patients and HCs

**Supplementary Table 2.** Significant differences in Rich-club properties between CD and HCs

**Supplementary Table 3.** Significant differences in nodal DC and G1 between CD and HCs

**Supplementary Table 4.** Significant differences in distribution of gradient scores in Sub-networks between CD patients and HCs

**Supplementary Table 5.** Significant effective connectivity associated with anxiety (HAD-A) in CD patients

**Supplementary Table 6.** Post-hoc linear regression and interaction analyses of DC values in CD patients

**Supplementary Table 7.** Significant differences in global properties and rich club between CD patients and HCs based on Parcellation 2 (final analytic sample, n=161)

**Supplementary Table 8.** Significant differences in nodal DC between CD and HCs based on Parcellation 2 (final analytic sample, n=161)

**Supplementary Table 9.** Demographic and clinical characteristics of CD patients and HCs in the demographically matched subsample (n=122)

**Supplementary Table 10.** Sensitivity analyses of global properties and rich-club organization: sex-stratified subsamples (males, n = 113; females, n = 48) and demographically matched subsample (n = 122), based on Parcellation 1

**Supplementary Table 11.** Sensitivity analyses of AUC of DC and Gradient score 1: sex-stratified subsamples (males, n = 113; females, n = 48) and demographically matched subsample (n = 122)

**Supplementary Sensitivity Analysis:** Validation of Group Differences against Methodological and Demographic Confounders.
